# The central molecular clock is robust in the face of behavioural arrhythmia in a *Drosophila* model of Alzheimer’s disease

**DOI:** 10.1242/dmm.014134

**Published:** 2014-02-26

**Authors:** Ko-Fan Chen, Bernard Possidente, David A. Lomas, Damian C. Crowther

**Affiliations:** 1 Department of Genetics, Downing Site, Cambridge, CB2 3EH, UK.; 2 Biology Department and Neuroscience Program, Skidmore College, Saratoga Springs, NY 12866, USA.; 3 Department of Medicine, University College London, London, W1T 7NF, UK.; 4 Cambridge Institute for Medical Research, Wellcome/MRC Building, Hills Road, Cambridge, CB2 0XY, UK.

**Keywords:** Alzheimer’s disease, Circadian dysfunction, Non-cell-autonomous Aβ toxicity, *Drosophila* model, Biological clock

## Abstract

Circadian behavioural deficits, including sleep irregularity and restlessness in the evening, are a distressing early feature of Alzheimer’s disease (AD). We have investigated these phenomena by studying the circadian behaviour of transgenic *Drosophila* expressing the amyloid beta peptide (Aβ). We find that Aβ expression results in an age-related loss of circadian behavioural rhythms despite ongoing normal molecular oscillations in the central clock neurons. Even in the absence of any behavioural correlate, the synchronised activity of the central clock remains protective, prolonging lifespan, in Aβ flies just as it does in control flies. Confocal microscopy and bioluminescence measurements point to processes downstream of the molecular clock as the main site of Aβ toxicity. In addition, there seems to be significant non-cell-autonomous Aβ toxicity resulting in morphological and probably functional signalling deficits in central clock neurons.

## INTRODUCTION

Alzheimer’s disease (AD) is the most common cause of dementia in adults and is characterised at the microscopic level by extracellular amyloid plaques and intraneuronal tau tangles. Amyloid plaques are composed of fibrillar aggregates of a spectrum of amyloid beta (Aβ) peptides derived from the proteolytic cleavage of amyloid precursor protein (APP) (LaFerla et al., 2007). The significance of Aβ is underpinned by the numerous disease-linked mutations that dysregulate APP processing: mutations that result in a spectrum of Aβ peptides with a higher aggregation propensity have been linked to familial AD (Philipson et al., 2010), whereas sequence variation in APP that reduces Aβ production is protective (Jonsson et al., 2012). There is much evidence from cell-culture and animal-model systems (Iijima-Ando and Iijima, 2010; Philipson et al., 2010) that the conformers of Aβ that possess neurotoxic activity are likely to be soluble oligomeric species rather than the more easily detected amyloid plaques (Lesné et al., 2006; Shankar et al., 2008; Ono et al., 2009; Tomic et al., 2009; Brorsson et al., 2010; Jo et al., 2011; Tang et al., 2012; Speretta et al., 2012).

Alongside the well-recognised memory and cognitive deficits that typify AD, a substantial proportion of individuals with AD also experience circadian abnormalities, including increased daytime napping, night-time restlessness and fragmented sleep. Taken together, these clinical features constitute a dampening of the variation in day-night activity (Volicer et al., 2001; Coogan et al., 2013); furthermore, two-thirds of individuals with AD that are living at home exhibit some degree of ‘sundowning’, in which restlessness and agitation increase late in the afternoon and early evening (Prinz et al., 1982; Volicer et al., 2001). It is readily apparent that such behavioural problems are a substantial burden for both AD individuals and their caregivers.

Circadian timekeeping in animals is a cell-autonomous mechanism based on the intrinsic 24-hour-period oscillation of ‘clock gene’ products (such as PER1, PER2, CRY1, CRY2, CLOCK and BMAL1 in humans) mediated by interlocked transcriptional-translational feedback and feedforward loops (TTFLs). Such cellular circadian oscillators are present throughout the body, but those in the suprachiasmatic nucleus (SCN; ~20,000 ‘clock neurons’) of the hypothalamus are considered to be the master pacemaker in humans (Mohawk et al., 2012). The SCN neurons are divided into a dorsal shell [arginine vasopressin (AVP)-positive] and ventral core [vasoactive intestinal polypeptide (VIP)-positive areas]. Circadian oscillators in the SCN are entrained by light to keep them in synchrony with the external light-dark cycle. The SCN then converts the entrained circadian signal into coordinated physiological and behavioural outputs via multiple humoural and neuronal pathways (Mohawk et al., 2012). Importantly, circadian oscillations are self-sustaining at both molecular and behavioural levels. Therefore ‘free-running’ rhythms continue even in the absence of external cues [e.g. the constant darkness (DD)].

The behavioural abnormalities linked to AD in the clinic have been substantiated by histological changes in the SCN in postmortem brains, in particular the cell loss observed by Swaab and colleagues (Swaab et al., 1985; Swaab et al., 1988). Despite the cell loss seen in the SCN in AD brains, amyloid plaques here are sparse (Coogan et al., 2013), possibly indicating that Aβ toxicity is largely non-cell-autonomous, being derived from neighbouring cells. Concordant with this, Tate and colleagues reported reduced amplitude of behavioural rhythms in rats carrying SCN grafts of PC12 cells expressing a disease-linked variant of APP as compared with animals grafted with control PC12 cells (Tate et al., 1992). However, subsequent murine studies of AD-linked circadian locomotor abnormalities, using established model systems, has yielded a complex and sometimes contradictory picture. In particular, mice expressing mutant APP in light-dark (LD) conditions exhibit normal circadian locomotor activity (Wisor et al., 2005; Ambrée et al., 2006; Gorman and Yellon, 2010). By contrast, increased locomotor activity during resting light hours was detected in transgenic animals expressing additional mutated human γ-secretase (APP×PS1) (Duncan et al., 2012) or the combination of mutant PS1 and tau (APP×PS1×tau) (Sterniczuk et al., 2010). Furthermore, only minor deficits in free-running behaviour (DD) are detected in these AD model systems (Wisor et al., 2005; Gorman and Yellon, 2010; Sterniczuk et al., 2010). For these reasons, the role of toxic Aβ species in circadian deficits in AD remains elusive.

**TRANSLATIONAL IMPACT**

**Clinical issue**

Alzheimer’s disease (AD) is the commonest cause of dementia in adults. At the microscopic level, AD is characterised by two main pathologies: firstly, extracellular amyloid plaques, which are composed of amyloid beta peptide (Aβ), derived from the proteolytic cleavage of amyloid precursor protein, and secondly, intraneuronal tau tangles. At the clinical level, alongside memory deficits, abnormalities in the sleep-wake cycle are an early feature of AD. Circadian rhythmicity in humans is controlled by a molecular clock in the central clock neurons in the suprachiasmatic nucleus (SCN) of the hypothalamus. Postmortem studies suggest that the loss of cells in the SCN contributes to circadian abnormalities in AD. However, it is not known whether the clock itself is degraded or whether communication of the rhythm to the periphery is lost in disease. A better understanding of the pathological mechanisms underlying circadian abnormalities in AD would facilitate the design of effective interventions that could improve well-being and clinical outcomes in individuals with AD, and their carers.

**Results**

*Drosophila* that express toxic isoforms of Aβ in the nervous system have previously been established as a model of AD. Here, the authors show that the pan-neuronal expression of Aβ in the brains of flies results in progressive loss of circadian behavioural rhythmicity, despite ongoing normal oscillations of the central molecular clock. Circadian deficits were most marked when Aβ was expressed in neighbouring neurons and glia rather than in the clock neurons themselves, and one target for this non-cell-autonomous Aβ toxicity seems to be the paracrine communication of the clock neurons. Finally, the authors demonstrate that entrainment of the central molecular clock by exposure to regular light-dark cycles, even in the face of behavioural arrhythmia, prolongs the flies’ lifespan.

**Implications and future directions**

This work shows clearly that, in a fly model of AD, the central molecular clock is robust in the face of behavioural arrhythmicity and that, despite having no observable influence on behaviour, an entrained clock is able to prolong life. These findings support the use of light therapy to entrain the clocks of individuals with, or at risk of, AD even if such an intervention produces no obvious behavioural response. Moreover, the discovery of a robust invertebrate model of non-cell-autonomous Aβ toxicity provides a platform for looking for ways to modulate this toxicity. The achievement of such a goal could have wide-ranging consequences for our understanding of AD that extend beyond circadian biology.

As a complement to murine models of AD, we have generated a *Drosophila* system to study Aβ toxicity. Instead of replicating the proteolytic processing of APP, we and others have fused the Aβ peptide with a secretion signal peptide and driven its expression in the nervous system (Finelli et al., 2004; Iijima et al., 2004; Crowther et al., 2005). Various Aβ species were expressed pan-neuronally in *Drosophila* using the *Gal4-UAS* expression system (Brand and Perrimon, 1993), and Aβ toxicity was detected using a range of biochemical, neuron-histological and behavioural assays (e.g. Jahn et al., 2011; Speretta et al., 2012; Huang et al., 2013). In this study we have combined the tools available to neurodegeneration modelling in the fly with the well-developed systems that are also available for studying circadian rhythms. The use of the fly as a model organism is justified by the many orthologies between *Drosophila* and human, in particular by the conserved circadian TTFLs, involving the clock genes *period*, *timeless*, *clock* and *cycle* (Allada and Chung, 2010). Circadian locomotor activity in *Drosophila* is controlled by ~150 clock-gene-expressing neurons (clock neurons) in the brain. As with the SCN in humans, *Drosophila* clock neurons can be divided into several groups (termed sLNvs, lLNvs, LNds, DN1s, DN2s, DN3s and LPNs in the fly) according to their ventral-dorsal anatomy and neuropeptide identity. Similar to the role of the neuropeptide VIP in synchronising among clock neurons in the SCN (Hastings and Herzog, 2004; Aton et al., 2005; Maywood et al., 2006), the neuropeptide PDF (pigment disperse factor), released from about 16 ventral neurons (sLNvs and lLNvs) in *Drosophila*, maintains robust circadian behaviour by paracrinely synchronising the molecular oscillation of clock neurons (e.g. Renn et al., 1999; Peng et al., 2003; Cusumano et al., 2009). In addition, the majority of the axons from these clock neurons project to the dorsal protocerebrum (dorsal commissure) (Helfrich-Förster et al., 2007), where they communicate with each other and to their downstream targets. Normal free-running circadian behaviour in *Drosophila* also requires correct signalling at these synapses (Kaneko et al., 2000; Blanchardon et al., 2001; Nitabach et al., 2002). Rezával et al. (Rezával et al., 2008) previously demonstrated that overexpression of wild-type human APP in PDF-positive ventral clock neurons (*pdf >hAPP*) resulted in age-dependent loss of circadian rhythm. Although *Drosophila* does have the γ-secretase required to process APP, it has little β-secretase-like (dBACE) activity and so the generation of Aβ peptides is inefficient (Fossgreen et al., 1998; Carmine-Simmen et al., 2009). Therefore, the circadian abnormality in *pdf >hAPP* flies (Rezával et al., 2008) is probably unrelated to toxic Aβ peptides. In this study, however, we have employed well-established tools for characterising the *Drosophila* clock system to investigate the mechanism of Aβ-mediated disruption of circadian rhythms.

## RESULTS

### Ubiquitous neuronal Aβ expression causes circadian behavioural deficits

**Fig. 1.**
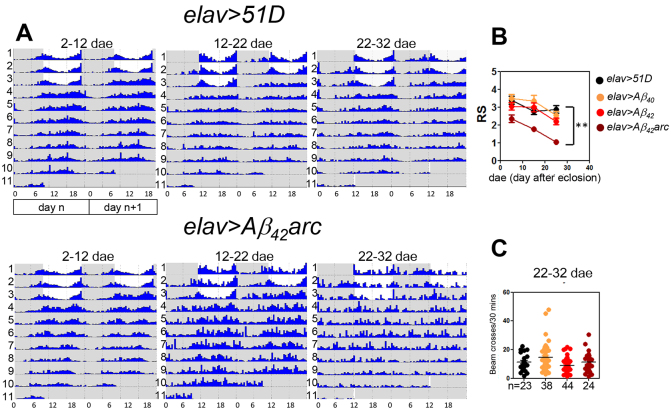
**Loss of circadian locomotor behaviour in Aβ_42_arc-expressing flies.** (A) Representative average actograms of 16 individuals at different age groups are shown for male controls (*elav>51D*, upper panels) and flies expressing Aβ_42_ arctic mutant (Aβ_42_arc) in all neurons (*elav>Aβ_42_arc*, lower panels). Two continuous days are plotted in each row (day *n* and day *n*+1; i.e. day *n*+1 in row 1 is equivalent to day *n* in row 2), in which the *y*-axis is activity in each day and *x*-axis is the hours in each day (the white area marks the light phase; the grey shaded area marks the dark phases). The ages of the flies are indicated as day after eclosion (dae). Numbers 1–11 represent actual days under recording. (B) Reduced periodicity, determined by average rhythmic statistic (RS; mean ± s.e.m.), is found in Aβ_42_arc-expressing flies compared with controls and flies expressing Aβ_40_ or Aβ_42_ peptide. Asterisks mark significance (*P*<0.01) by non-parametric one-way ANOVA between *elav>Aβ_42_arc* and *elav>51D* controls. (C) No difference in average locomotor activity (i.e. mean of beam crosses) are found among indicated genotypes in the age group of 22–32 dae. Number of flies tested is indicated.

To determine whether our Aβ-expressing flies (Crowther et al., 2005; Jahn et al., 2011) exhibit disturbed intrinsic circadian rhythms, we monitored their circadian locomotor activities in constant darkness (DD). By calculating autocorrelation coefficients we quantified the robustness of their circadian periodicity [arrhythmia is defined as rhythmic statistic (RS) ≤1.5] (Levine et al., 2002). The *Gal4-UAS* system was used to drive expression from a single transgene of each of Aβ_40_, Aβ_42_ and the arctic (E22G) variant of the Aβ_42_ peptide in the *Drosophila* nervous system (*elav>Aβ_40_*, *elav>Aβ_42_* and *elav>Aβ_42_arc*; Fig. 1). While still young [2–12 days after eclosion (dae) and 12–22 dae], the Aβ_40_ and Aβ_42_ flies exhibited robust circadian rhythmicity in DD that was essentially identical to that observed in control flies (*elav>51D*). Although a subpopulation of Aβ_40_- and Aβ_42_-expressing flies developed arrhythmic behaviour by the age of 22–32 dae, their average RS did not differ significantly from controls (Table 1). By contrast, the overall rhythmicity of flies expressing pan-neuronal Aβ_42_arc was significantly reduced compared with controls at all age groups (RS; Table 1). Furthermore, there was an age-related progression in dysrhythmia with a significant decline in the RS between the 2–12 dae and 22–32 dae groups (Table 1). At 22–32 dae, about 80% of the *elav>Aβ_42_arc* flies were arrhythmic, whereas the majority of the age-matched controls flies remained rhythmic (Table 1). Consistent with the age-dependent decline in circadian behaviour, the arrhythmic pattern in the averaged actogram of *elav>Aβ_42_arc* flies was clear by 22–32 dae (Fig. 1A). Although all flies tested exhibited age-related decline in circadian rhythmicity (Fig. 1B), those expressing Aβ_42_arc were significantly worse than other genotypes. The appearance of discreet Aβ deposits, akin to plaques, in Aβ_42_arc fly brains (supplementary material Fig. S1A–E) correlates with the severity of behavioural disruption. However, the amount of such large Aβ aggregates in the brain does not predict behavioural arrhythmia because, for any given Aβ genotype, there is no difference in plaque density between behaviourally rhythmic and arrhythmic flies (supplementary material Fig. S1F and see Discussion).

**Table 1.**
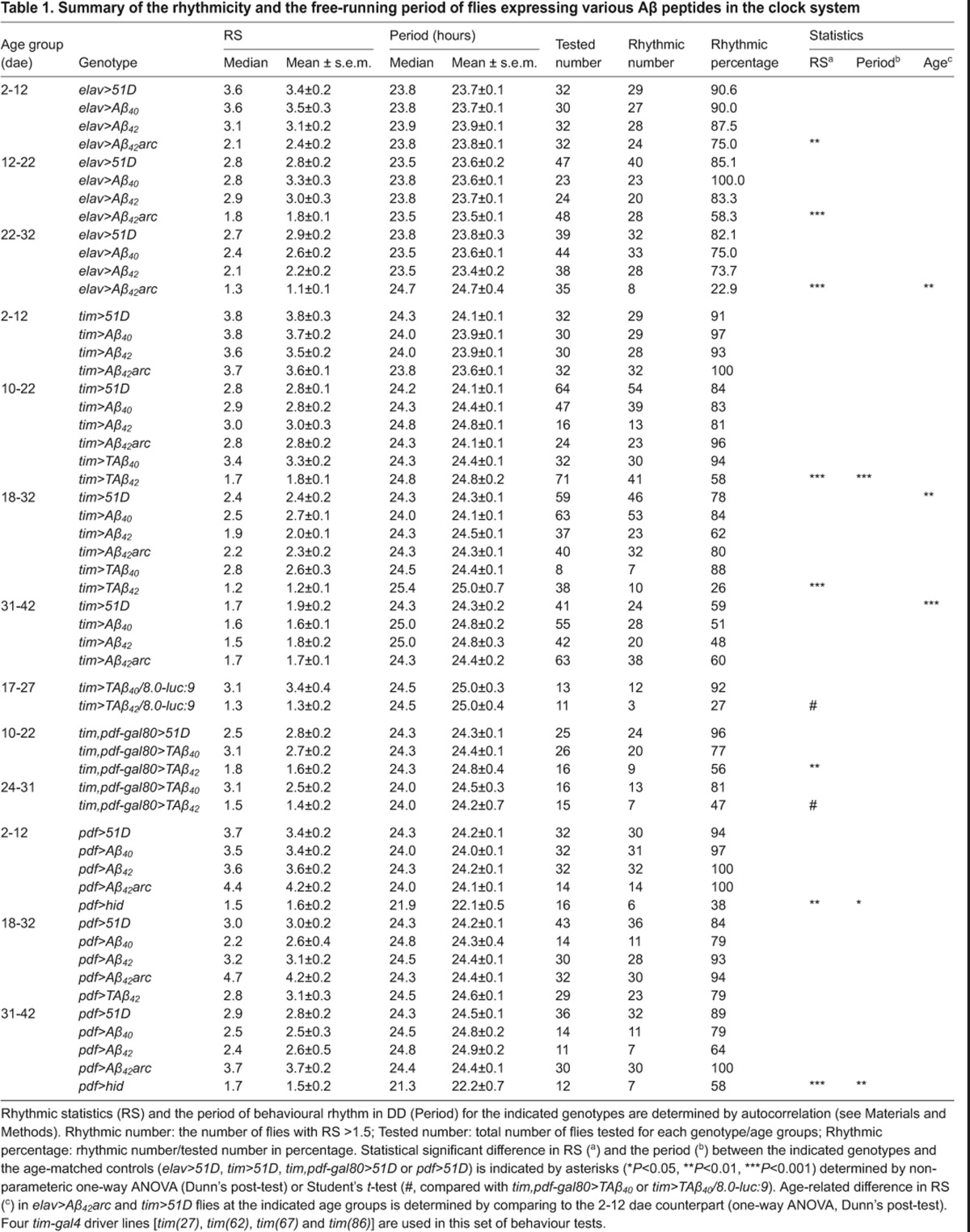
Summary of the rhythmicity and the free-running period of flies expressing various Aβ peptides in the clock system

To exclude the possibility that the age-related decline in walking velocity in Aβ-expressing flies (Jahn et al., 2011) was confounding our observations of circadian rhythmicity, we assessed the overall daily locomotor activity. When we counted the number of beam crosses in our DAM apparatus, there were no significant differences between any genotypes at least until age 22–32 dae (Fig. 1C). This indicates that the loss of circadian activity is not a function of overall decreased locomotor activity.

### Once arrhythmia develops in DD it cannot be reversed by LD

**Fig. 2.**
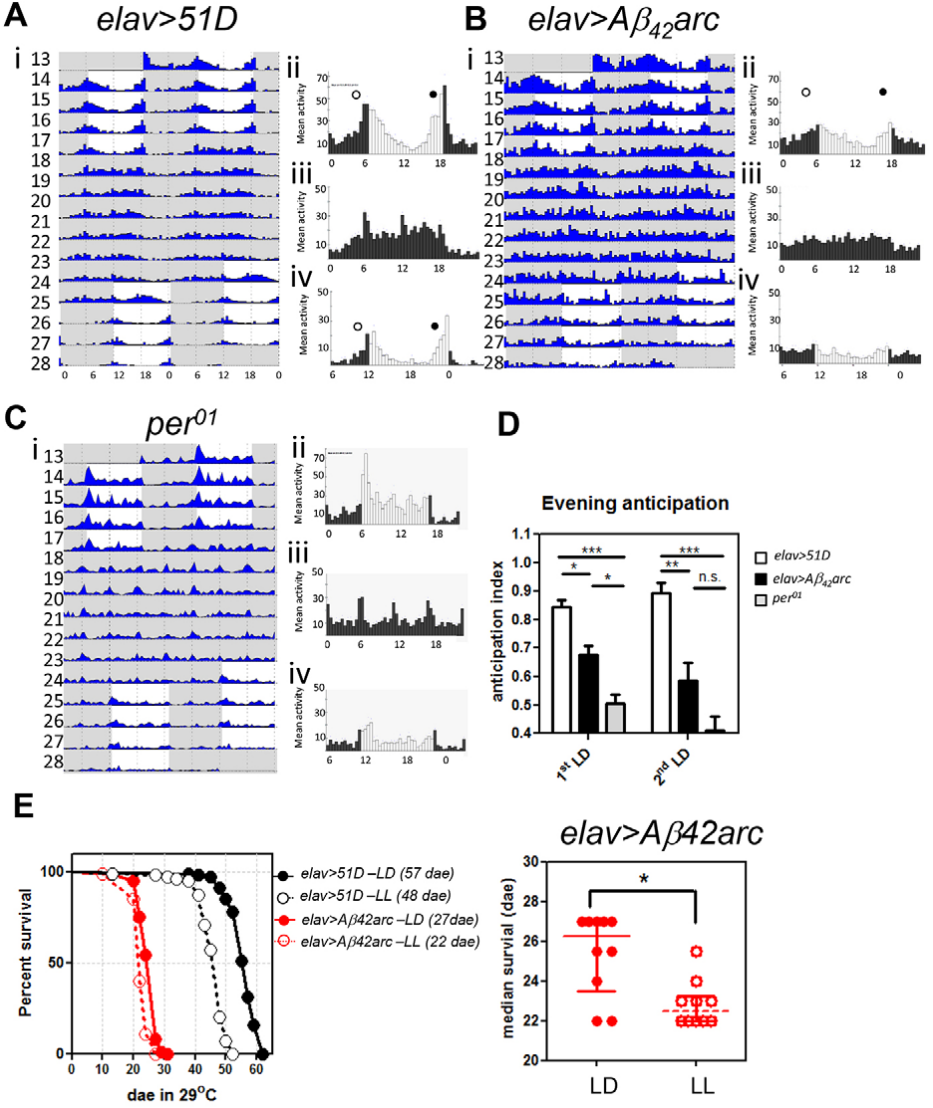
**Arrhythmia in Aβ_42_arc-expressing flies is not reversible by LD cycles.** (Ai) Average actogram of control flies (age 14 dae, *n*=16) undergoing 4 days LD, 6 days DD and followed by a 6-hour phase-shifted LD for 4 days. Two continuous days are plotted in each row (day *n* on the left and day *n*+1 on the right; i.e. day *n*+1 in row 13 is equivalent to day *n* in row 14), in which the *y*-axis is activity in each day and *x*-axis is the hours in each day; the grey shaded area marks the dark phases. Numbers 13–28 represent dae under recording. (Aii-iv) The average daily activity histogram for the first 4 days LD (ii), 6 days DD (iii) and the final 4 days LD (iv) are plotted. Black bars: dark phase; white bars: light phase; morning ramping: white circles; evening ramping: black circles. (B) The average actogram (i) and histograms (ii-iv) of arrhythmic *elav>Aβ_42_arc* flies (age 14 dae and arrhythmic during DD, *n*=19). (C) *per^01^* flies are used as negative controls, showing no anticipation. (D) Quantification of the evening anticipatory activity (mean ± s.e.m.) for the first LD and second LD shown in A-C by anticipatory index (the total activity 3 hours before darkness/the total activity 6 hours before darkness). Asterisks: significant difference by non-parametric one-way ANOVA (***P<0.001; **P<0.01; *P<0.05; n.s.: not significant). (E) Left panel: survival curves under LD (solid line) or LL (dashed lines) for *elav>Aβ_42_arc* (red line) or *elav>51D* (black line) flies. The estimated median survivals (dae) from 100 individuals in each genotype are indicated in brackets. Flies in LL die significantly sooner that those in LD (*P*<0.001, log-rank test) in both *elav>51D* and *elav>Aβ_42_arc*. Right panel: a more conservative measure in median survival is calculated from the ten sets of ten *elav>Aβ_42_arc* flies in LD (filled circle) and LL (blank circles). Bars indicate median and first and third quartiles. Asterisk: significance determined by non-parametric Student’s *t*-test (*P*<0.05).

Because of the characteristic dampening of circadian rhythm in individuals with AD, light treatment has been used in an attempt to enhance circadian rhythmicity (Coogan et al., 2013). To investigate the reversibility of the circadian arrhythmia in Aβ-expressing flies, we identified young flies that displayed arrhythmic behaviour when transferred from LD to DD. We then returned them to LD, with a 6-hour shift in the cycle, and looked to see whether rhythmic behaviour was restored. Flies with an intact circadian clock respond not only to the actual light changes in LD with a startle reflex but also anticipate dawn and dusk with 2–3 hours of increased locomotor activity (Stoleru et al., 2005; Lim et al., 2007). We used this circadian controlled ‘anticipatory ramping’ in activity as the marker of rhythmicity in these experiments. In control flies (*elav>51D*) exposed to LD, anticipatory ramping in behaviour was seen, as expected, before dawn (white circle, Fig. 2A) and dusk (black circle, Fig. 2A). In continuous darkness, control flies retained robust circadian behaviour, with one peak of activity during the 24-hour period (Fig. 2Aiii). In the following secondary LD cycles, control flies were able to re-synchronise with the new phase within 1 day (Fig. 2Ai,iv). Under the same conditions, *elav>Aβ_42_arc* flies retained substantial rhythmic behaviour during the first LD cycles (Fig. 2Bi,ii) but became arrhythmic in the following constant darkness (cf. Fig. 1A; Fig. 2Bi,iii). Furthermore, *elav>Aβ_42_arc* flies largely failed to re-synchronise and exhibited weak ramping activity in the secondary LD (Fig. 2Bi,iv). The Harrisingh anticipatory index was used to objectively quantify the degree of entrainment in *elav>Aβ_42_arc* flies during LD (Harrisingh et al., 2007). We found that *elav>Aβ_42_arc* flies have a much reduced anticipatory index as compared with the controls, and they were comparable to the non-anticipatory *period*-null mutants (*per^01^*) during the secondary LD cycles (Fig. 2Aiv,Biv,Civ,D). Taken together, we found that none of the DD-arrhythmic *elav>Aβ_42_arc* flies were able to regain vigorous rhythmicity during the subsequent LD cycles, indicating that behavioural deficits are not readily remediated by re-exposure to a rhythmic 24-hour LD cycle.

### The LD environment benefits arrhythmic Aβ-expressing flies

Having established that DD-arrhythmic flies could not be substantially re-entrained by subsequent exposure to an LD environment, we were interested to know whether, despite this, a rhythmic environment could still provide benefits for an arrhythmic organism. To investigate this possibility, we took control and Aβ_42_arc-expressing flies and compared their longevity in LD and continuous light (LL) conditions. Consistent with a previous study (Pittendrigh and Minis, 1972), there was a significant reduction in the lifespan for control flies in LL (48 dae *elav>51D*, Fig. 2E) as compared with LD (57 dae, *elav>51D*, *P*<0.001, log-rank test, *n*=100, as 10 sets of 10 flies, for each condition, 18% increase over LL, Fig. 2E). Remarkably, Aβ_42_arc-expressing flies benefited identically from LD despite their arrhythmic behaviour (*elav>Aβ_42_arc*, LL: 22 dae vs LD: 27 dae, *P*<0.001, log-rank test, *n*=100 for each conditions, 18% increase over LL and *P*<0.05; Student’s *t*-test, 10 sets of 10 flies, Fig. 2E). Because LL conditions disrupt both behavioural and molecular circadian oscillations in flies (Konopka et al., 1989; Marrus et al., 1996), the observed increase in lifespan on going from LL to LD might be the result of residual clock functions in *elav>Aβ_42_arc* flies despite their behavioural arrhythmicity.

### The central molecular clock continues to oscillate in Aβ-expressing flies

**Fig. 3.**
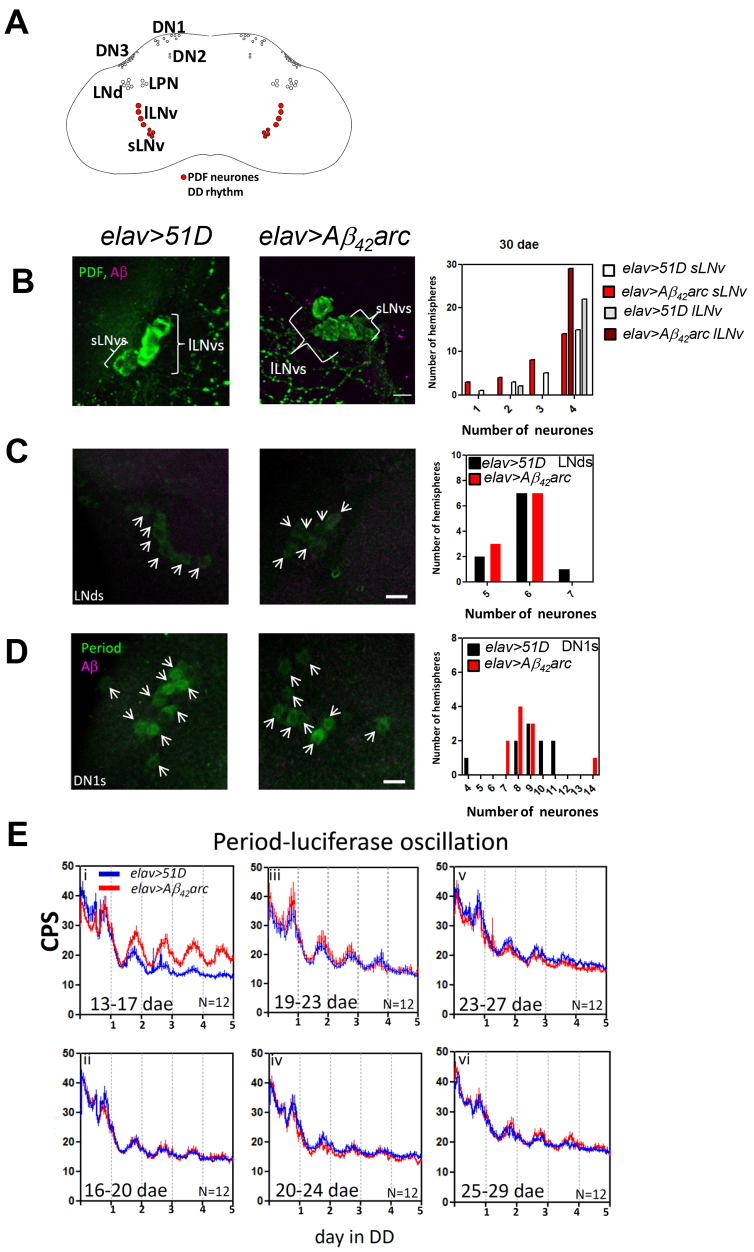
**Intact central molecular clock in *elav>Aβ_42_ar*c flies.** (A) Scheme showing the 150 central clock neurons divided into seven groups. Red circles: PDF neurons, essential for DD rhythm. (B) Left panels: immunostaining of PDF neurons (sLNvs and lLNvs; green) and Aβ (magenta) for control *elav>51D* and *elav>Aβ_42_arc* flies. Right panels: no difference (determined by χ^2^-test) was found in the number of PDF neurons between *elav>Aβ_42_arc* (*n*=29 brain hemispheres) and control *elav>51D* (*n*=24) flies. (C,D) Dorsal clock neurons, LNds (C) and DN1s (D), are identified by Period (Per)-positive staining (green) and anatomical localisation (arrows) for 30-dae-old *elav>51D* and *elav>Aβ_42_arc* flies. No difference (by χ^2^-test) in the number of LNd and DN1 neurons were found between *elav>Aβ_42_arc* (*n*=15, 30 dae) and control *elav>51D* (*n*=13, 30 dae). Magenta: Aβ peptide. Scale bars: 10 μm. (E) None of the pairwise differences between amplitudes of *8.0-luc:9* profiles in *elav>Aβ_42_arc* flies (red, mean ± s.e.m.) and that in *elav>51D* controls (blue, mean ± s.e.m.) are significant at indicated age groups (see supplementary material Table S1 for detailed statistics). As the experiment was designed as continuous sets of overlapping 5-day windows on *8.0-luc:9* luciferase activity profile, Kruskal-Wallis ANOVA statistics (with Dunn’s Multiple Comparison post-test), instead of multiple *t*-test, are used to account for variation and to determine the significance of differences in amplitude as compared with age-matched controls. Numbers of flies are indicated (N). CPS, counts per second.

We used two approaches to test, at both the cellular and molecular levels, whether the central clock apparatus remains intact during the progression of Aβ toxicity even though the flies exhibit arrhythmic behaviour. The first approach involved the direct visualisation of the structural integrity of a subgroup of clock neurons (PDF-positive cells) that are essential for maintaining intrinsic rhythmicity in DD (scheme of clock neurons, Fig. 3A) (Renn et al., 1999). When we compared control flies and Aβ_42_arc-expressing flies by counting the number of PDF-positive cell bodies, we found no differences at least until 30 dae (Fig. 3B). We also visualised two dorsal neuronal groups – LNds and DN1s – by staining for Period (Per) protein. Again, we found no evidence of clock neuron loss in *elav>Aβ_42_arc* flies as compared with controls (Fig. 3C,D). Additionally, there was no correlation between the Aβ-plaque density and the number of clock neurons in *elav>Aβ_42_arc* fly brains (supplementary material Fig. S1G). Despite the absence of gross structural changes in the clock neurons, we employed a second approach to look for functional deterioration. By measuring the bioluminescence derived from a Per-luciferase fusion construct, *8.0-luc:9* (Veleri et al., 2003), we were able to monitor the molecular clock as it pertains to Per protein oscillation. Previous work has indicated that the *8.0-luc:9* strain faithfully reports molecular clock oscillations in non-PDF dorsal clock neurons (DNs and LNds) in the fly brain (Hodge and Stanewsky, 2008; Sekine et al., 2008; Sehadova et al., 2009). Comparing *8.0-luc:9/elav>Aβ_42_arc* flies with equivalent control flies that do not express Aβ (Fig. 3E), we found that the rhythmic robustness of the bioluminescence was the same for both populations. To confirm that the bioluminescence from the Per-luciferase fusion construct was correctly reporting the oscillation of Per protein, we immunostained the brains of control and *Aβ_42_arc*-expressing flies for Per at various times during the second day of constant darkness post-entrainment. By specifically assessing the intensity of Per staining in the clock neurons (DN1s, LNds and sLNvs) we could confirm that the behaviourally arrhythmic *Aβ_42_arc*-expressing flies exhibited the same diurnal Per oscillation as we see in control flies (green staining, supplementary material Fig. S2). By contrast, there was no circadian variation in the density of the Aβ peptide deposits (magenta, supplementary material Fig. S3A).

Taken together, these data indicate that the circadian behavioural abnormalities seen in Aβ-expressing flies are not caused by loss of central molecular clock function but are more likely due to damage in the downstream pathways in the clock neurons or other distal brain region.

### Aβ_42_ expression in clock cells is insufficient to cause circadian arrhythmicity

**Fig. 4.**
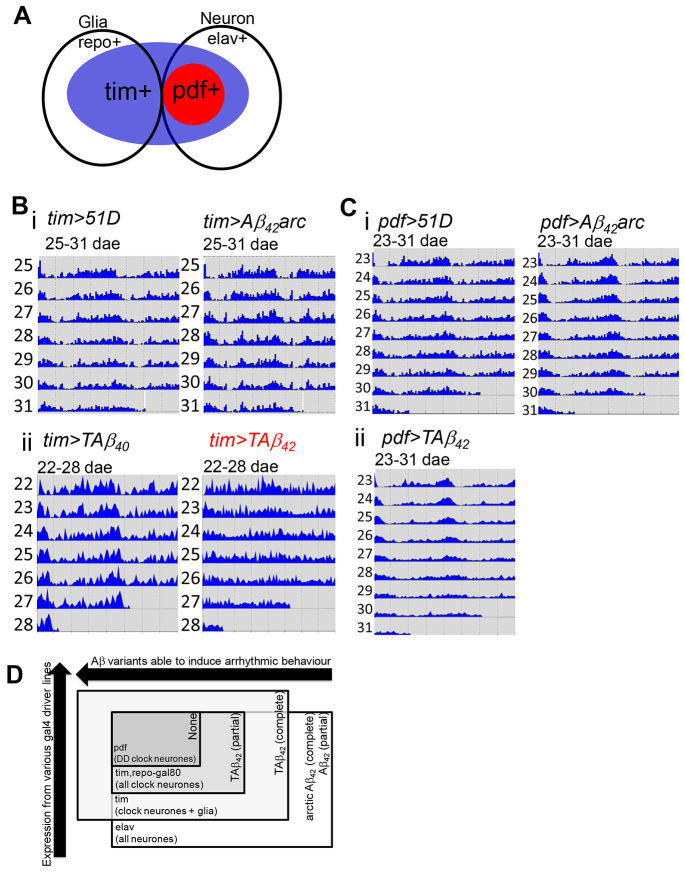
**Clock neurons are relatively resistant to Aβ_42_ toxicity.** (A) Scheme showing *gal4* and *gal80* transgene expression in *elav-gal4* (elav+, all neurons), *pdf-gal4* (pdf+, red, PDF-positive neurons) and *tim-gal4* (tim+, blue, including neurons and glia) driver lines, and *repo-gal80* (repo+, all glia) and *pdf-gal80* (pdf+, red, PDF positive neurons) repressor lines in the *Drosophila* nervous system. (Bi,Ci) Average actograms showing normal DD rhythms in *tim>Aβ_42_arc* (*n*=16, 25–31 dae) and *pdf>Aβ_42_arc* (*n*=16, 23–31 dae) as compared with *tim>51D* (*n*=16, 25–31 dae) and *pdf>51D* (*n*=15, 23–31 dae). (Bii,Cii) Average actograms showing arrhythmic DD behaviour in *tim>TAβ_42_* (*n*=12, 22–28 dae) and normal rhythm in *pdf>TAβ_42_* (*n*=11, 23–31 dae) as compared to control *tim>TAβ_40_* (*n*=8, 22–28 dae) and *pdf>51D* (i, *n*=15, 23–31 dae; see Ci). See Table 1 for the total number of flies tested. (D) Scheme summarising that circadian arrhythmia derived from Aβ_42_ expression within various extent centred on clock neurons in relation to two factors: the Aβ_42_ species required for causing behavioural arrhythmia (horizontal: right to left including Aβ_42_, Aβ_42_arc and TAβ_42_ species) and the extent of Aβ_42_ expression (vertical, bottom to top with more restricted expression). PDF neurons (dark grey rectangle) and all clock neurons (light grey rectangle) are more resistant to Aβ_42_ insults, because TAβ_42_ triggers no (dark grey rectangle) or intermediate (light grey rectangle) circadian arrhythmia, whereas TAβ_42_ in all clock cells and Aβ_42_arc expression in all neurons is enough to cause complete arrhythmia (white rectangle).

Although these results underline the importance of Aβ-mediated degradation of the clock output pathways, the studies showing cell loss in the SCN in AD postmortem samples indicate that damage to the central clock neurons is a possible cause for circadian rhythm deficits (Wu and Swaab, 2007). For this reason we were interested to know how the flies would respond to a range of Aβ insults that were restricted to the clock system. To this end we used the driver lines *timeless-gal4*, which drives expression in all clock cells including neurons and glia (Fig. 4A), and *pdf-gal4*, which drives expression in PDF-positive clock neurons (Kaneko and Hall, 2000). As with *elav-gal4*, the clock-neuron-specific expression of the less-aggregation-prone Aβ isoforms Aβ_40_ and Aβ_42_ had few or no behavioural consequences. Surprisingly, both *pdf>Aβ_42_arc* and *tim>Aβ_42_arc* expression exhibited robust behaviour; this is in contrast to the arrhythmicity induced when Aβ_42_arc was expressed ubiquitously (cf. Fig. 4Bi,Ci and Fig. 1A). To test the possibility that the absence of any phenotype is due to lower expression levels for *pdf* and *tim* lines, as compared to *elav*, we measured gal4-dependent expression of GFP as a control. When we quantified GFP fluorescence specifically in PDF-positive neurons we found that the expression levels were essentially identical for all three (*elav*, *pdf* and *tim*) drivers (arrows in supplementary material Fig. S4A,B and quantification in S4C). The only remarkable difference was that, for *elav*-driven expression, the levels of GFP in the bulk of the brain (that is the non-clock neurons) were higher, as expected (supplementary material Fig. S4D). Taken together, these data indicate that Aβ expression that is restricted to clock cells is insufficient to trigger circadian abnormalities.

### More focused expression of Aβ in clock neurons promotes further resistance to circadian arrhythmia

Although Aβ_42_arc expression in clock cells was compatible with normal circadian behaviour, we tested whether the highly toxic tandem Aβ_42_ construct (TAβ_42_) could induce arrhythmia under similar conditions. TAβ_42_ consists of two repeats of the Aβ_42_ sequence linked by a glycine-rich 12mer linker peptide. We have previously shown that TAβ_42_ has an oligomer-rich aggregation mechanism (Speretta et al., 2012) and indeed *elav*-driven expression at 25°C results in developmental lethality (data not shown). By contrast, the TAβ_40_ variant, although equally aggregation prone, does not form oligomers and is essentially non-toxic (Speretta et al., 2012). When TAβ_42_ was expressed in all clock cells using the *tim* driver, we observed significant arrhythmic behaviour as compared with *tim>Aβ_42_arc* and non-Aβ controls (ages between 10 dae and 32 dae; Table 1 and Fig. 4Bii). Further restriction in the scope of TAβ_42_ expression was achieved by using *repo-gal80* to suppress expression in the glial subset of tim-positive cells (*tim,repo-gal80*, supplementary material Fig. S5; Fig. 4A). Although more rhythmic than the *tim>TAβ_42_* flies at the same age, *tim,repo-gal80>TAβ_42_* flies showed a reduced rhythmic percentage and reduced rhythmicity as compared to controls (supplementary material Fig. S5B) without any potentially confounding decrease in average locomotor activity (supplementary material Fig. S5C). Remarkably, even TAβ_42_, when driven by *pdf-gal4*, was insufficient to induce arrhythmic behaviour (Fig. 4Cii; Table 1), indicating that the roles of Aβ expression outside PDF neurons are likely to be of primary importance. Moreover, cell-autonomous effects of Aβ are likely to be a relatively minor contributor to circadian abnormalities (Fig. 4D).

### The molecular clock continues to oscillate until the neurons die

**Fig. 5.**
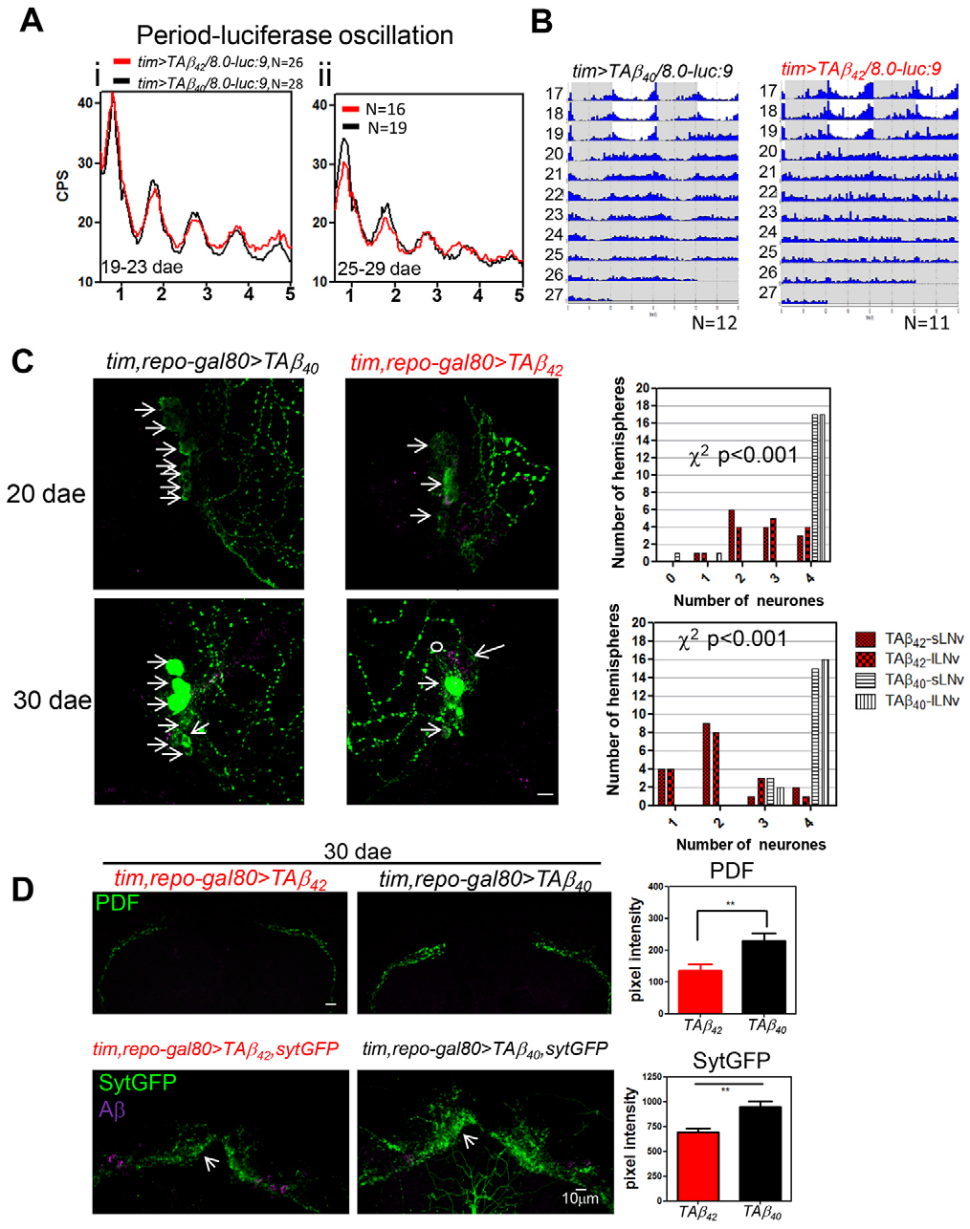
**TAβ_42_ expression results in dysfunction and loss of clock neurons and has a minor effect on the remaining molecular clock.** (A) Similar bioluminescence profiles (Per-luciferase fusion, *8.0-luc:9*) are found for *tim>TAβ_42_/8.0-luc:9* (red, average trace) and control *tim>TAβ_40_/8.0-luc:9* (black, average trace) between ages 19 dae to 29 dae. The profiles represent data merged from several flies (*n* from 16 to 28 per experiment). CPS, counts per second. (B) Average actograms for *tim>TAβ_42_/8.0-luc:9* (arrhythmic) and control *tim>TAβ_40_/8.0-luc:9* flies. Two continuous days are plotted in each row (day *n* on the left and day *n*+1 on the right; i.e. day *n*+1 in row 17 is equivalent to day *n* in row 18), in which the *y*-axis is activity in each day and *x*-axis is the hours in each day; the grey shaded area marks the dark phases. Numbers 17–27 represent dae in recording. (C) Image stacks showing that fewer PDF neurons (green and arrows) coincided with Aβ-positive staining (magenta and circle) in *tim,repo-gal80>TAβ_42_* fly brains as compared with those in *tim,repo-gal80>TAβ_40_* flies at the indicated age groups. Significant differences (χ^2^-test) in the number of sLNv and lLNvs were found between the two genotypes (*P*<0.001). Number of hemispheres *n*=16 for 30 dae, *tim,repo-gal80>TAβ_42_*; *n*=18 for 30 dae, *tim,repo-gal80>TAβ_40_*; *n*=14 for 20 dae, *tim,repo-gal80>TAβ_42_*; *n*=18 for 20 dae, *tim,repo-gal80>TAβ_40_*. (D) Top panels: image stacks demonstrating reduced PDF peptide signal (green) at dorsal termini of sLNvs in 30-dae-old *tim,repo-gal80>TAβ_42_* fly brain as compared with controls. The PDF signals in *tim,repo-gal80>TAβ_42_* (red bars, mean ± s.e.m.) are significantly lower than that in *tim,repo-gal80>TAβ_40_* (black bars, mean ± s.e.m., **P<0.01, Student’s *t*-test). Number of hemispheres (*n*)=16 for *tim,repo-gal80>TAβ_42_*; *n*=18 for *tim,repo-gal80>TAβ_40_*. Lower panel: dorsal output axonal terminal (arrows) of clock neurons is marked by synaptotagmin-GFP (sytGFP, green). Significant reduction in sytGFP signal in 30-dae-old *tim,repo-gal80>TAβ_42_,sytGFP* flies (red bars, mean ± s.e.m.) was observed as compared with *tim,repo-gal80>TAβ_40_,sytGFP* controls (black bars, mean ± s.e.m.) at ZT3 (**P<0.01, Student’s *t*-test). ZT denotes zeitgeber time with ZT0 indicating dawn and ZT12 dusk during LD cycles. *n*=14, *tim,repo-gal80>TAβ_42_,sytGFP*; 8, *tim,repo-gal80>TAβ_40_,sytGFP*. Aβ signal (magenta). Scale bars: 10 μm.

The preceding data indicate that pan-clock expression, driven by *tim-gal4*, is the most clock-restricted domain that generates robust TAβ_42_-mediated circadian arrhythmia. To assess the functional and structural integrity of the clock system under these conditions we again employed a number of molecular and cellular techniques. In *tim>8.0-luc:9/TAβ_42_* flies, aged between 19 dae and 29 dae, we found that the central molecular clock retained its rhythmicity (Fig. 5A) despite the behavioural arrhythmia and significant clock-cell loss induced by expression of TAβ_42_ (Fig. 5B; supplementary material Fig. S6). A similar pattern of behavioural arrhythmia and cell loss was observed when TAβ_42_ was restricted to clock neurons (*tim,repo-gal80>TAβ_42_*, Fig. 5C; supplementary material Fig. S5).

We also studied Aβ_42_-linked synaptic dysfunction in clock neurons in two ways using confocal microscopy; firstly, we immunostained the brains for the PDF peptide, as a marker of PDF-positive clock neurons, and, secondly, we assessed the intensity of a chimaeric GFP-synaptotagmin construct that accumulates presynaptically in all clock neurons. In both cases we quantified the signal intensity at the dorsal termini, a major site of clock neuron axonal projection. We found that the presence of TAβ_42_ markedly reduced PDF peptide and GFP signals in these areas, indicating a paracrine abnormality in PDF neurons and presynaptic dysfunction in clock neurons (Fig. 5D). Given the relatively robust character of the molecular clock signal in the face of extensive neuronal dysfunction, and appreciable neuronal death, it seems likely that the central molecular clock continues to ‘tick’ until the cells die and are physically lost. In other words, the molecular clock seems to be the most robust feature of the clock system during Aβ pathology, being interrupted only by neuronal dysfunction and death.

### Non-cell-autonomous Aβ toxicity

**Fig. 6.**
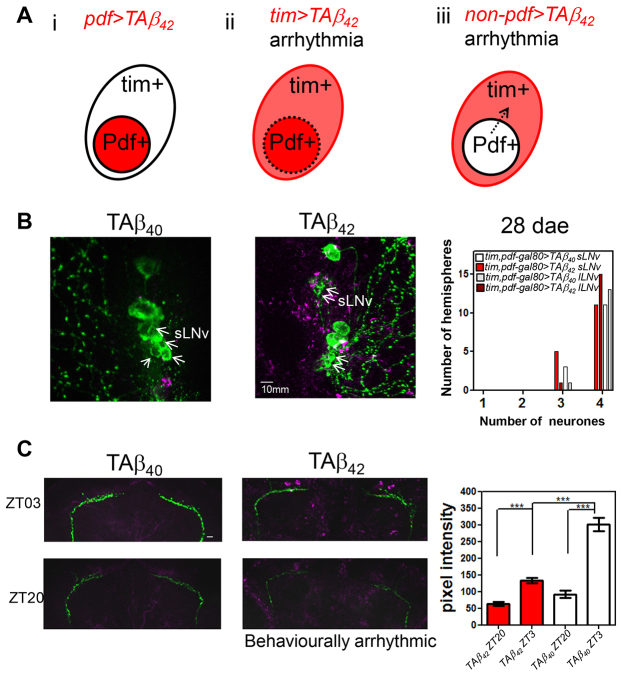
**TAβ_42_-mediated non-cell-autonomous toxicity to PDF neurons.** (A) Schemes showing that: (i) expression of TAβ_42_ (red area, *pdf>TAβ_42_*) in only PDF neurons results in no circadian abnormality up to 30 dae; (ii) expression of TAβ_42_ in all clock cells (red and pink areas, *tim>TAβ_42_*) results in circadian arrhythmia and loss of PDF neurons (marked by dashed outline of PDF neurons); (iii) expression of TAβ_42_ exclusively in clock cells except for PDF neurons (pink area, *non-pdf>TAβ_42_*) results in no neuronal loss but reduced PDF peptide signal (dashed arrow) in PDF neurons. (B) Left panels: representative images demonstrating no PDF neuronal loss (green) in *tim,pdf-gal80>TAβ_42_* as compared with control *tim,pdf-gal80>TAβ_40_*. Aβ plaques are detected in *tim,pdf-gal80>TAβ_42_* fly brains (magenta). No difference in the number of PDF neurons for both sLNvs (arrows) and lLNvs was detected between 28-dae *tim,pdf-gal80>TAβ_42_* and *tim,pdf-gal80>TAβ_40_* (see graph on right). Significance was determined by χ^2^-test. See Table 1 for reduced behavioural rhythmicity in *tim,pdf-gal80>TAβ_42_*. (C) Left panels: representative image stacks demonstrating reduced PDF peptide signal at dorsal termini of sLNvs in *tim,pdf-gal80>TAβ_42_* fly brain as compared with controls at indicated time points during LD cycles. ZT denotes zeitgeber time with ZT0 indicating dawn and ZT12 dusk during LD cycles. Right panels: PDF signals in *tim,pdf-gal80>TAβ_42_* (red bars, mean ± s.e.m.) are significantly lower than that in *tim,pdf-gal80>TAβ_40_* (white bars, mean ± s.e.m.) at ZT3 (***P<0.001, one-way ANOVA). Although both genotypes maintain the daily oscillation of PDF peptide signal (ZT3 vs ZT20, ***P<0.001, one-way ANOVA), no oscillation of Aβ plaque density was detected (see supplementary material Fig. S3B). *n*=16, *tim,pdf-gal80>TAβ_42_*; 14, *tim,pdf-gal80>TAβ_40_*. Scale bar: 10 μm.

So far we have demonstrated that even TAβ_42_ does not induce arrhythmia when expressed exclusively in PDF neurons (Fig. 6Ai); by contrast, use of a *tim-gal4* driver that includes glia, pdf neurons and other nearby clock neurons does cause arrhythmia (Fig. 6Aii). Therefore, we were interested to determine whether the behavioural disturbance consequent on the expression of Aβ is mediated by cell-autonomous mechanisms, or not. To achieve the expression of TAβ_42_ throughout the clock system but specifically not in the PDF-positive neurons that control the DD rhythm (Fig. 6Aiii), we used the *tim,pdf-gal80* driver. In this experiment we find that, although the number of PDF neurons is not affected by the expression of TAβ_42_, we do see a reduction in PDF peptide staining in the dorsal terminus of PDF-positive neurons (Fig. 6B). The hypothesis that PDF neurons are dysfunctional, despite not expressing Aβ themselves, was justified by the finding that the flies also exhibited behavioural arrhythmia in DD (Table 1).

## DISCUSSION

### Pan-neuronal Aβ expression causes circadian arrhythmia

The dampening, and eventual disintegration, of circadian behaviour in individuals with AD constitutes one of the most distressing clinical features of the disorder. Although there is evidence that cell loss in the SCN is correlated with such symptoms of AD, it is not known whether damage to the molecular clock, the clock neurons, or rather to the output pathway, underpins the behavioural deficits. To address these questions we have expressed Aβ peptides pan-neuronally in the fly brain and assessed the consequences for circadian behaviour. During the lifespan of our flies, we found that pan-neuronal Aβ_40_ and Aβ_42_ expression had no effect on circadian locomotor behaviour. By contrast, expressing the Arctic variant of Aβ_42_ (Aβ_42_arc) resulted in profound age-dependent behavioural arrhythmia as evidenced by a progressively increasing arrhythmic sub-population, reduced overall rhythm robustness in DD (Fig. 1) and loss of anticipatory activity in LD (Fig. 2), recapitulating the dampening of behavioural rhythms in individuals with AD (Volicer et al., 2001) and demonstrating a loss of circadian regulation that could be considered as the fly equivalent of ‘sundowning’ (Fig. 2D). Flies were not aged past 35 days because thereafter control flies exhibit circadian abnormalities (Rezával et al., 2008; Luo et al., 2012; Rakshit et al., 2012; Umezaki et al., 2012) and the differences between Aβ-expressing and control flies become less clear.

The more marked consequences of Aβ_42_arc expression are thought to be due to the increased aggregation propensity of this peptide and to its oligomer-rich aggregation mechanism (Nilsberth et al., 2001; Whalen et al., 2005; Luheshi et al., 2007). Our previous studies have shown that the fly reports the presence of such oligomeric aggregates and these species correlate more closely with climbing and longevity deficits than do large insoluble aggregates (Luheshi et al., 2007; Speretta et al., 2012). Likewise, the circadian behavioural abnormalities demonstrated here also correlate more closely with oligomer formation (Aβ_40_≤TAβ_40_≤Aβ_42_<Aβ_42_arc<TAβ_42_, see Figs 1 and 4) than they do with total Aβ plaque density in the fly brains (supplementary material Fig. S1). Therefore, we reason that oligomeric species instead of large aggregates are the likely cause of the circadian deficits. These findings are concordant with similar experiments in the mouse, where animals expressing a disease-linked APP mutation, with or without mutant PS1 and tau, were found to retain essentially normal DD circadian behaviour throughout life (Wisor et al., 2005; Sterniczuk et al., 2010). Because these mice represent a model of wild-type Aβ_42_ overproduction, this might explain why, like in the *elav>Aβ_42_* flies, little or no circadian abnormality was observed (Table 1). It is possible that increasing the levels of oligomeric aggregates, by introducing additional copies of the wild-type Aβ_42_ transgene, or by ageing the flies for longer, might also elicit circadian abnormalities. However, as it stands, our fly model of Aβ_42_arc toxicity is the first experimental animal that robustly recapitulates the progressive circadian deficits found in AD.

### Behavioural arrhythmia is not due to disruption of the molecular clock

One of the remarkable conclusions of our study is that central clock neurons survive and continue to exhibit circadian oscillation of at least one clock-related protein in the face of Aβ-induced behavioural arrhythmia. This was most apparent in flies expressing pan-neuronal arctic Aβ_42_ (Aβ_42_arc). Despite exhibiting locomotor arrhythmia from ~12 dae, these flies had morphologically normal central clock neurons until at least 30 dae. Specifically, the PDF-positive sLNv neurons, which are essential for DD rhythmicity, remained intact. Furthermore, the non-PDF clock neurons continued to express the Per-luciferase reporter construct (*8.0-luc:9*) in a circadian pattern that was essentially identical to rhythmic control flies (Fig. 3E). Concordant with this finding, immunostaining of Per protein in Aβ_42_arc-expressing flies also confirmed its normal circadian oscillation in clock neurons during constant darkness and despite Aβ-induced arrhythmia (in DN1s, LNds and sLNvs; supplementary material Fig. S2). When we subsequently restricted the neuronal expression of Aβ to the clock system using *tim-gal4* we observed equivalent behavioural arrhythmia; however, in this context, Aβ_42_arc was insufficiently potent. Instead, the highly toxic tandem Aβ_42_ construct was required to induce arrhythmia and was accompanied by some loss of both sLNv and ILNv neurons. Despite the expression of this highly toxic Aβ construct, and the consequent loss of PDF neurons, the molecular oscillations in dorsal clock neurons continued unabated (Fig. 5). Taken together, our data indicate that cell-autonomous Aβ toxicity is insufficient to disrupt the oscillation of the central molecular clock.

### Aβ_42_ expression in clock neurons is insufficient to cause circadian arrhythmicity

Notable was the lack of a circadian phenotype even when we expressed the highly toxic TAβ_42_ in PDF neurons alone. Indeed, the resistance of the PDF neurons to Aβ_42_ expression has been remarked upon in passing by others (DiAngelo et al., 2011). When we expanded the expression of TAβ_42_ to include all clock neurons, by using *tim,repo-gal80*, the flies became behaviourally arrhythmic. Indeed, further expansion to also include tim-positive glial cells (using *tim-gal4*), resulted in a progressive circadian degradation in TAβ_42_ flies and so supports a role for glia in modulating clock neuronal activity (Ng et al., 2011). Such evidence highlights the contribution of circuits peripheral to the central clock neurons in mediating Aβ-linked circadian locomotor deficits, particularly in the ageing brain (Nakamura et al., 2011; Luo et al., 2012).

Our conclusions are concordant with Pallier and colleagues, who studied the R6/2 Huntingdon’s disease (HD) mouse (Pallier et al., 2007) and found that a Per-luciferase reporter continued to oscillate in *ex vivo* SCN preparations despite the behavioural arrhythmia of the donor animals. These investigators, like us, concluded that the primary target for circadian disruption in the R6/2 mouse is external to the central pacemaker. Such parallels between AD and HD models indicate that damage to the communication between central pacemaker neurons and other neuronal circuits might be a pathological feature that is common to neurodegenerative disorders. In the human context, the central clock neurons in the SCN are thought to use the pineal gland as one of their downstream targets; in this regard it is interesting to note that one consequence of AD is that the secretion of melatonin from the pineal becomes arrhythmic (Wu et al., 2006).

### The axons of the clock neurons are a target for Aβ toxicity

The manifestation of circadian locomotor rhythmicity in the fly requires the synchronisation of the molecular clocks in central clock neurons, downstream neurons and in the peripheral tissues. All this is thought to be dependent on both peptidergic paracrine and synaptic communication. Our work has particularly implicated dysfunction in the dorsal protocerebrum as an important pathological mechanism, on the basis of reduced PDF staining and synaptotagmin-GFP (sytGFP) intensities in this area (Fig. 5D). When determining the mechanisms underlying Aβ toxicity on pacemaker PDF neurons, we have demonstrated that PDF peptide signal is reduced by expressing Aβ not in PDF neurons themselves but in neighbouring cells that are in communication with PDF neurons (i.e. *tim,pdf-gal80>TAβ_42_*, Fig. 6Aiii). Such non-cell-autonomous toxicity of Aβ has long been suspected (Busche et al., 2008); however, to our knowledge, this is the first *in vivo* demonstration of toxicity in one neuron as a consequence of Aβ being expressed explicitly by its neighbours. Considering the importance of PDF peptides in synchronising clock neurons under DD conditions, such reduced PDF signals likely contribute to the behavioural arrhythmia in *tim,pdf-gal80>TAβ_42_* flies. Furthermore, the cell-autonomous toxicity of Aβ on PDF neurons likely had no role in generating these circadian phenotypes (Fig. 6A).

Nevertheless, Aβ-mediated arrhythmia cannot be equated to a pure loss of the PDF signal, because killing PDF neurons by expressing hid (*pdf>hid*, Table 1) results in characteristic short period rhythms in the remaining rhythmic subpopulation, something that we do not see in any of our Aβ-expressing flies. Notably, the behavioural abnormalities in *tim>TAβ_42_* and *tim,pdf-gal80>TAβ_42_* flies resembles the phenotype of *tim>tetanus-toxin*, in which synaptic blockade results in arrhythmic flies despite an intact molecular clock (Kaneko et al., 2000). In summary, our findings signify Aβ-mediated damage to both axonal outputs and paracrine signalling in clock neurons.

### An entrained central clock benefits the organism despite behavioural arrhythmia

It has previously been documented that animals in a rhythmic LD environment live longer than those exposed to rhythm-disrupting light cycles (Pittendrigh and Minis, 1972; Davidson et al., 2006; Park et al., 2012). Similarly, loss of normal circadian behaviour in humans is associated with increased morbidity and mortality (Paudel et al., 2010; Tranah et al., 2011). Of particular interest recently has been the finding that behavioural and molecular arrhythmia in *per^01^* flies is accompanied by increased oxidative stress (Krishnan et al., 2012) and this could increase neurotoxicity in AD (Rival et al., 2009). However, it is unclear whether it is the circadian behaviour pattern per se, or alternatively an entrained molecular clock, that prolongs life. In this regard we have made the interesting observation that profoundly arrhythmic Aβ_42_arc flies live longer when exposed to LD as compared to those in the clock-disrupting LL environment. Indeed, the proportional increase in median survival is identical for both control and arrhythmic Aβ flies on going from LL to LD. In the absence of a visible behavioural correlate, it seems likely that an entrained molecular clock is beneficial and it is not the behavioural rhythms that prolong life.

Nevertheless, our data agree with the findings of Park and colleagues who concluded that the harmonious interaction of endogenous and environmental rhythms is optimal for longevity, something that was lost in their *per1per2* double-null mice (Park et al., 2012). Much is still unclear though; for example, we do not know whether entrainment needs to be central, or whether entrainment of one or more peripheral tissue clocks is sufficient. We can also speculate that the harmonious interplay of endogenous rhythms and behavioural activity might have an important role in protecting the organism from the oxidative stress that is a key feature of both arrhythmic organisms and individuals with AD. In particular, the circadian variation in antioxidant proteins such as peroxiredoxins (O’Neill et al., 2011; Edgar et al., 2012) might be timed to best protect the organism from the stress of oxidative cellular metabolism. Indeed it is notable that our arrhythmic Aβ-expressing flies show a general dampening in circadian behaviour, being moderately active throughout a 24-hour cycle. By contrast, *per^01^* flies, which have a genetically impaired molecular clock, seem to respond to the light phase with activity and to dark with relative inactivity (Fig. 2Civ), being entirely arrhythmic only in continuous dark. Comparing the oxidative consequences of behavioural arrhythmia in these two contexts could provide interesting insights into pathogenesis of AD.

Nevertheless, these findings have implications for the environment that we provide for individuals with AD; indeed, it is already established that good circadian light hygiene can result in circadian behavioural improvements (Coogan et al., 2013). By contrast, our work has shown that, once Aβ-expressing flies became arrhythmic in a dark environment, light-dark cycling can no longer significantly restore circadian behaviour patterns. Despite this, our work has indicated that the benefits of a clean light-dark environment might not be expressed as improved behavioural endpoints. Our research points to potentially disease-modifying benefits of an entrained molecular clock, possibly as a consequence of reduced oxidative damage, something that is known to characterise AD from its earliest stages (Nunomura et al., 2001; Markesbery et al., 2005).

## MATERIALS AND METHODS

### Fly strain and husbandry

All *gal4* and *gal80* lines expressing in the *Drosophila* clock system are gifts from Prof. Ralf Stanewsky (Queen Mary, London, UK), including *tim-gal4 (27)*, *tim-gal4 (62)*, *tim-gal4 (67)*, *tim-gal4 (86)*, *tim-gal4 (27),pdf-gal80* and *pdf-gal4* (Kaneko and Hall, 2000; Chen et al., 2011). Various *UAS-Aβ* lines, including *UAS-Aβ_40_*, *UAS-Aβ_42_*, *UAS-Aβ_42_arc*, *UAS-TAβ_40_* and *UAS-TAβ_42_*, were previously generated by the site-specific Phi31C system using acceptor line *51D* (therefore as the background control) and backcrossed to *w^1118^* (Jahn et al., 2011; Speretta et al., 2012). The acceptor site is marked by RFP; however, we recently noticed that 20% of our *w^1118^*;*51D* line lost this signal during backcrossing. The pan-neuronal expression of Aβ is driven by *elav-gal4^c155^* (Crowther et al., 2005). The fly strain, *tim,repo-gal80*, expressing Gal4 exclusively in *timeless* clock neurons, were generated by combining two transgenes: *tim-gal4 (67)* and *repo-gal80(N18)* (Awasaki et al., 2011). The *UAS-synaptotagmin-GFP* fly strain is a gift from Dr Cahir O’Kane (Department of Genetics, University of Cambridge). All the flies are reared in cornmeal food vials at 25°C and 70% humidity with continuous light-dark cycles (14 hour:10 hour).

### Locomotor behaviour assay

The *Drosophila* circadian locomotor assay is adapted from that described previously (Chen et al., 2011). No more than 20 adult male flies of each genotype are aged in a cornmeal food, replaced every other day, before being transferred individually to a glass tube containing 2% w/v agar and 5% w/v sucrose. The age of the flies is expressed as the day after eclosion (dae) in this study. One-dimensional locomotor activity of each individual fly is then detected continuously by summing the beam crosses every 30 minutes in an automated infrared beam monitoring system (DAM system, Trikinetics, Waltham, USA). The DAM apparatus is placed in a temperature-regulated incubator (Model 200, LMS Ltd, UK), in which the light condition is regulated by a compact fluorescent lamp (660 lumen, Eveready, UK) controlled by an external 24-hour timer.

For detecting intrinsic circadian locomotor rhythm, flies at a given age are first entrained by 3 days of 12 hour:12 hour light-dark cycles (LD cycles) followed by 7 days of constant darkness (DD) at 25°C. The overall level of locomotor activity for individual flies was calculated by averaging the beam crosses/30 minutes over the 10-day duration of the experiment. The time-series of daily activity (actogram) for each fly is plotted and analysed using the Flytoolbox in MATLab software (Levine et al., 2002). The free-running period of the individual time-series under constant darkness is determined by an autocorrelation base method (Levine et al., 2002), in which the rhythmicity statistic (RS) value is derived as a measure of rhythmic robustness. The RS value is the ratio of the autocorrelation coefficient value of an activity time-series to its 95% confidence interval of sampling error (Levine et al., 2002). The mean and median of the RS values are calculated for all tested genotypes (Table 1). All flies with an RS value ≤1.5 are classed as arrhythmic (Levine et al., 2002; Chen et al., 2011). The rhythmic percentage is the fraction of flies that achieve an RS >1.5. The mean and median of the period (hour) are calculated for each genotype from all rhythmic individuals. D’Agostino and Pearson omnibus normality test (GraphPad) was used to verify the normality of each dataset in this study before using parametric statistics; otherwise, non-parametric statistics have been applied. Non-parametric one-way ANOVA (Kruskal-Wallis with Dunn’s comparison post-test) or Student’s *t*-test are used to analyse differences in rhythmicity (RS) and the period between various genotypes and age groups (GraphPad software, Prism).

To assess whether LD rhythm can be re-entrained following DD, 14-dae *elav>Aβ_42_arc* and *elav>51D* flies are first entrained by 4-day LD cycles, followed by 6 days of DD and a secondary series of four LD cycles with a 6-hour phase delay as compared with the primary LD. Flies with an RS value ≤1.5 are defined as arrhythmic during DD. The average actogram are plotted by Flytoolbox for all LD and DD sessions. In LD, the anticipatory activity ramps at dark-light (morning) and light-dark (evening) transitions are visualised using histograms plots. Quantification of the evening anticipation is chosen because of a clear difference in evening ramping activity between *elav>51D* flies and the arrhythmic *period*-null mutants (*per^01^*) (see Stoleru et al., 2004) and also it is the fly equivalent of the ‘sundowning’ behaviour that is particularly significant for individuals with AD (Volicer et al., 2001). The anticipation quantification is based on Harrisingh/Individual Index (Harrisingh et al., 2007) by calculating the ratio of the total activity during the 3 hours before light-dark (evening) transitions to those in the 6 hours before the transitions for the first (2–4 day) and second (12–14 day) LD cycles.

### Longevity assay

Flies containing UAS-Aβ variants are crossed with *elav-gal4^c155^* driver lines. Female progeny are collected on the day of eclosion and mated for 24 hours before rearing in either 12-hour LD cycles or constant light (LL) at 29°C. The longevity was analysed as described previously (Crowther et al., 2005) with each assay being comprised of ten tubes of ten flies each (total 100 flies for each genotype). The statistical significance in median survival between LL and LD conditions was determined in two ways: (1) by using the estimates from the 100 individuals with the log-rank test (*n*=100) and, (2) more conservatively, by using the non-paired Student’s *t*-test for ten population median survival derived from the ten tubes of ten flies for each conditions. Statistical significance was set at *P*<0.05.

### Luciferase assay

Luciferase assays are modified from Chen et al. (Chen et al., 2011). Briefly, male flies expressing Per-luciferase protein fusion, *8.0-luc:9* (Veleri et al., 2003), and Aβ variants were generated from crossing *elav-gal4;8.0-luc:9* (this study) or *8.0-luc:9;tim-gal4* [obtained from Stanewsky (Hodge and Stanewsky, 2008)] to *UAS-Aβ* strains. Flies of each genotype are loaded in a white 96-well microtiter plate containing 5% w/v sucrose, 1% w/v agar and 15 mM luciferin (L-8220, Biosynth AG, Switzerland). Bioluminescence emitted from the flies was measured in a Packard Topcount Multiplate Scintillation Counter at 25°C for 2–3 days of LD before entering DD. Data were plotted and analysed using BRASS Version 2.1.3 (Locke et al., 2005). Fast Fourier transform-non-linear least squares (FFT-NLLS) was also performed by BRASS to estimate the period and the relative amplitude of each luciferase time-series. Relative amplitude error (Rel-amp error) values are used as a measure for rhythm robustness: if >0.7 then the individual luciferase activity would be assigned as arrhythmic (Stanewsky et al., 1997). Non-parametric one-way ANOVA are performed to analyse the significant difference (*P*<0.05) in the estimated period, amplitude and Rel-amp error among genotypes.

### Confocal immunohistochemistry

The immunohistochemistry protocol is modified from that previously described (Hermann et al., 2012). After 3 days entrainment in LD conditions, male flies of each genotype at the given age were fixed at the indicated ZT and CT (defined below) in 4% w/v paraformaldehyde/PB0.1%T (0.1 M phosphate buffer, pH 7.4 with 0.1% v/v Triton X-100) at room temperature for 2.5 hours. ZT denotes zeitgeber time with ZT0 indicating dawn and ZT12 dusk during LD cycles. CT denotes constant time, with CT00 indicating subjective dawn and CT12 subjective dusk. After fixation, the samples are washed three times with PB at room temperature (RT). The whole brain was dissected out and blocked with 10% v/v goat serum in PB with 0.5% v/v Triton X-100 (PB0.5%T) for 2 hours at RT and stained with monoclonal mouse anti-PDF (1:1000, PDFC7, Developmental Studies Hybridoma Bank, USA) and polyclonal rabbit anti-Aβ_1–16_ (1:500, SIG-39322, Covance) in PB0.5%T at 4°C for 48 hours. After washing six times in PB0.1%T, the samples are incubated at 4°C overnight with Alexa-Fluor-647-conjugated anti-rabbit and Alexa-Fluor-488-conjugated anti-mouse antibodies (Molecular Probes) diluted 1:300 in PB0.5%T. For Per and Aβ double staining (supplementary material Fig. S2), rabbit anti-Per (1:1000, gift from Ralf Stanewsky, QMUL) and monoclonal mouse anti-Aβ_1–16_ (1:500, 6E10, Covance) are used and the secondary antibodies are Alexa-Fluor-647-conjugated anti-mouse and Alexa-Fluor-488-conjugated anti-rabbit (Molecular Probes). Brains were washed six times in PB0.1%T before being mounted in Vectashield. Samples were stored at 4°C until examination under a Nikon Eclipse C1si confocal microscope.

### Quantification of confocal images

#### PDF neuron number

Image stacks are acquired along the anterior-posterior axis (*z*-axis) of the fly brain for each genotype from the confocal microscope with 40× magnification. ImageJ software was used to process and analyse all images. Large (lLNvs) and small (sLNvs) PDF neurons are identified by their anatomical location, size and PDF-peptide-positive staining. The number of the two neuron groups was counted separately per brain hemisphere (normally four per hemisphere of each neuronal group) (Helfrich-Förster et al., 2007) in the indicated genotype. The ratio of brain hemispheres with four PDF-positive neurons versus less than four was calculated. The significance of the differences was calculated using the χ^2^-test in GraphPad Prism.

#### PDF peptide signal at the dorsal terminus

The brightest PDF signal in the dorsal terminus of sLNvs in individual brains was identified across the *z*-axis by tuning signal gain around saturation. A single confocal image was then taken below signal saturation with the same laser intensity and signal gain across all samples of the indicated genotypes. The PDF signal (S_pdf_) was quantified by a ROI (region of interest) mask in ImageJ software. The same ROI mask was then moved to brain areas with no PDF signal to quantify the background signal (S_b_), which was then subtracted from S_pdf_. The average of S_pdf_ values, corrected in this way, was calculated for all individuals of the indicated genotypes and the difference among genotypes are determined by non-parametric one-way ANOVA.

#### GFP intensity

Image stacks containing PDF neurons are captured along the *z*-axis. The mean grey scale pixel intensities of GFP within PDF neurons were calculated by individual ROI circular masks outlined by the PDF-positive cell body (GFP_pdf_). The average GFP signal in the observed brain area (GFP_b_) for the indicated genotype was calculated from all the image stacks in a fixed field of view (318 μm^2^). Both GFP_pdf_ and GFP_b_ signals were documented and compared among genotypes by non-parametric one-way ANOVA.

## Supplementary Material

Supplementary Material
