# Supplementary material for: The central molecular clock is robust in the face of behavioural arrhythmia in a Drosophila model of Alzheimer’s disease
Source: Dis Model Mech. 2014 Feb 26;7(4):445–58. doi: 10.1242/dmm.014134 (PMC3974455; doi:10.1242/dmm.014134)
Supplement: Supplementary Material [file supp_7_4_445__index.html]

The central molecular clock is robust in the face of behavioural arrhythmia in a Drosophila model of Alzheimer’s disease — Supplementary Material 

# The central molecular clock is robust in the face of behavioural arrhythmia in a *Drosophila* model of Alzheimer’s disease

## DMM014134 Supplementary Material

**Files in this Data Supplement:**

- **Supplementary Material**
