# Supplementary material for: The central molecular clock is robust in the face of behavioural arrhythmia in a Drosophila model of Alzheimer’s disease
Source: Dis Model Mech. 2014 Feb 26;7(4):445–58. doi: 10.1242/dmm.014134 (PMC3974455; doi:10.1242/dmm.014134)
Supplement: Supplementary Material [file supp_7.4.445_DMM014134.pdf]

Figure S1

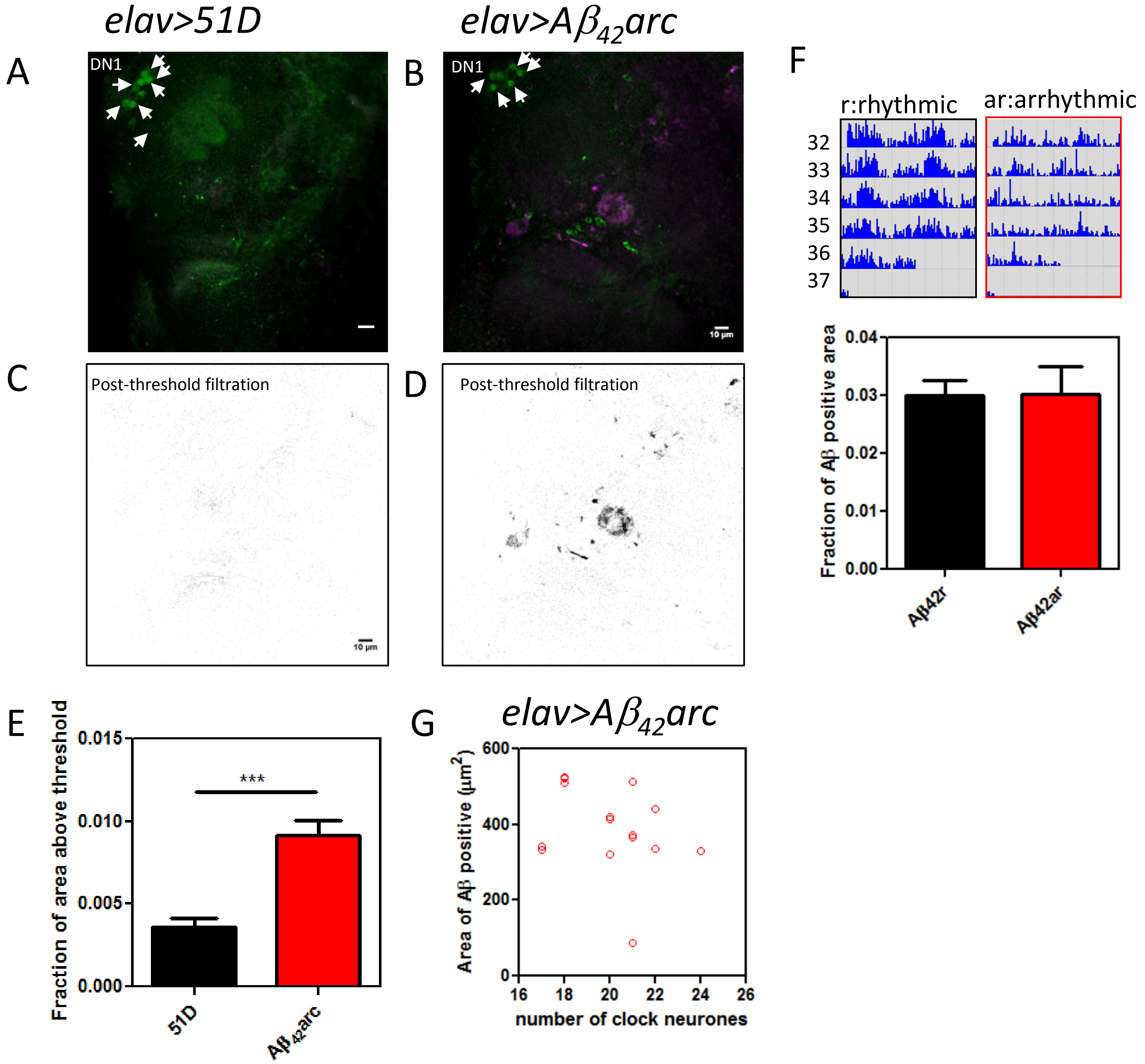

elav>51D
elav>$A\beta_{42}$arc
A
B
C
D
E
F
G
DN1
DN1
10 µm
Post-threshold filtration
Post-threshold filtration
10 µm
r:rhythmic
ar:arrhythmic
32
33
34
35
36
37
Fraction of Aβ positive area
0.04
0.03
0.02
0.01
0.00
Aβ42r
Aβ42ar
Fraction of area above threshold
0.015
0.010
0.005
0.000
***
51D
$A\beta_{42}$arc
elav>$A\beta_{42}$arc
Area of Aβ positive (µm^2)
600
400
200
0
16
18
20
22
24
26
number of clock neurones

Figure S2

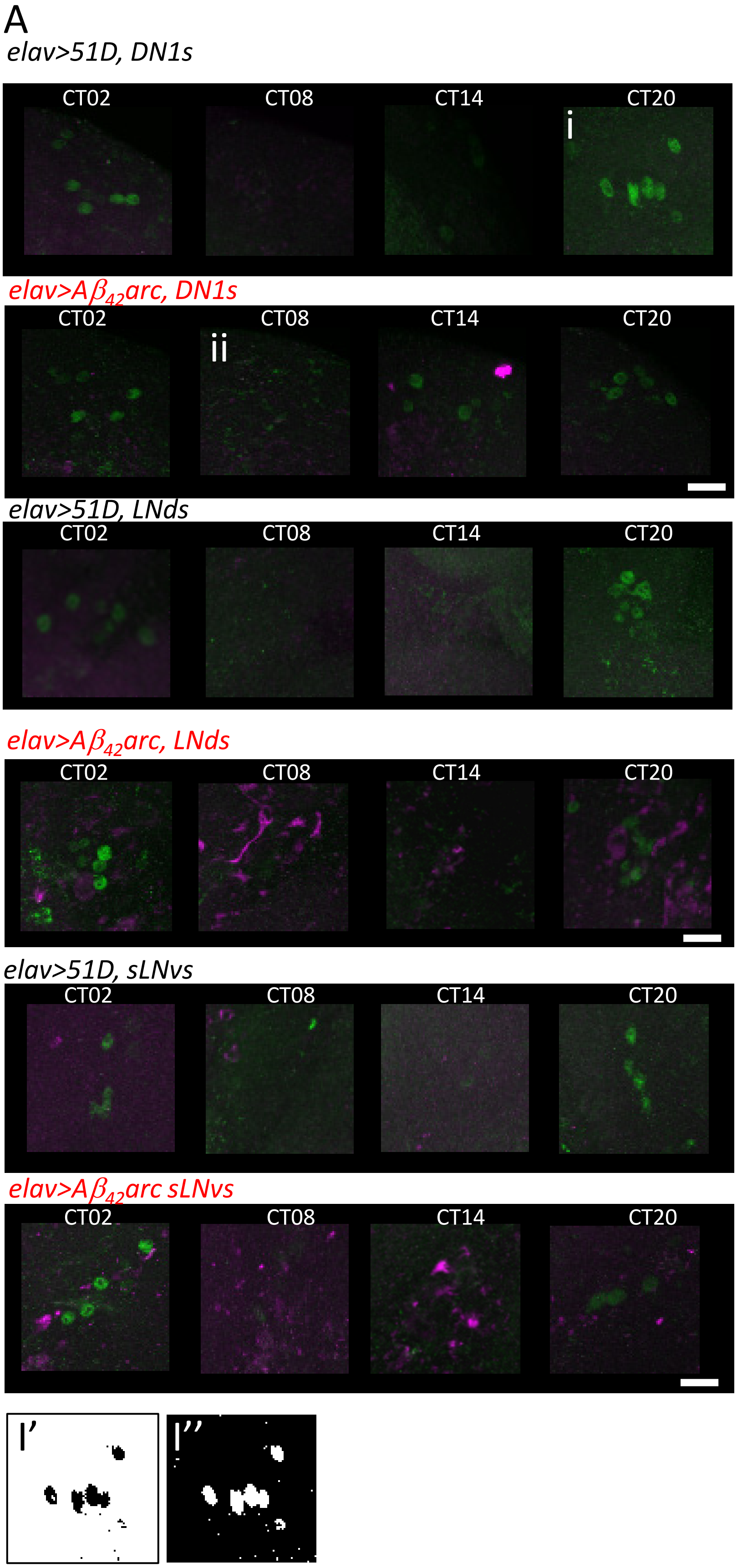

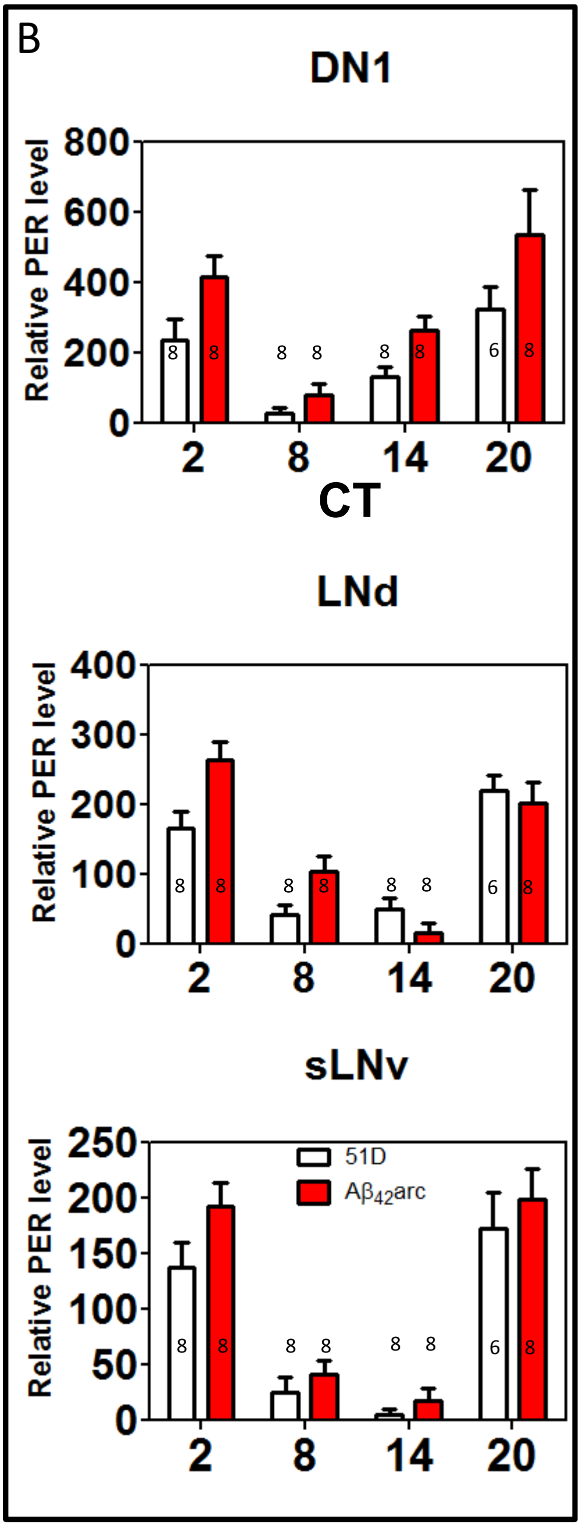

Mi'= mean intensity in the black area (Area.i')
Mi''= mean intensity in the black area (Area.i'')
Relative Period level= Area.i' x (Mi'-Mi'')/ Mi''

Figure S3

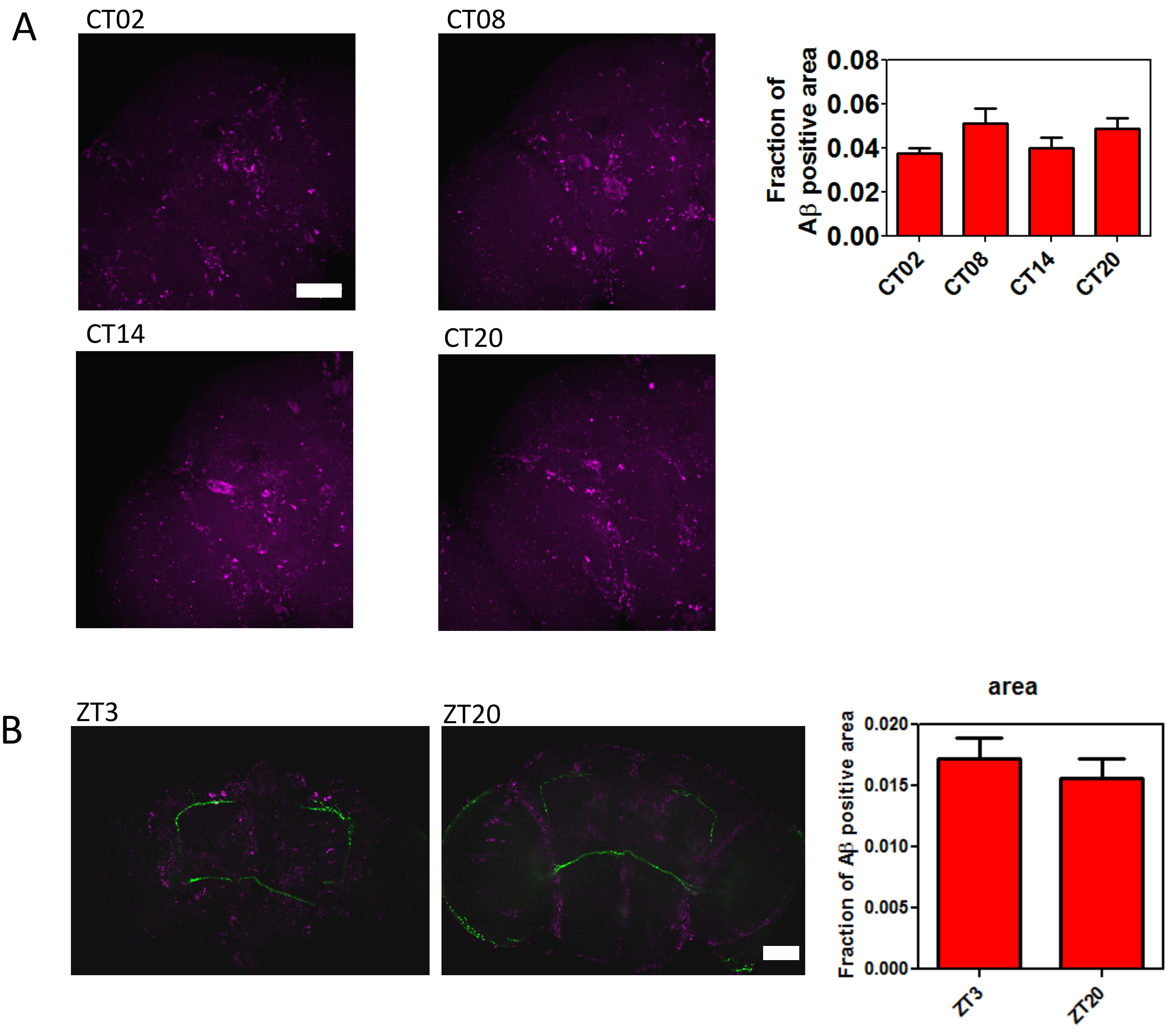
A
CT02
CT08
CT14
CT20
Fraction of
Aβ positive area
0.08
0.06
0.04
0.02
0.00
CT02
CT08
CT14
CT20
B
ZT3
ZT20
area
Fraction of Aβ positive area
0.020
0.015
0.010
0.005
0.000
ZT3
ZT20

Figure S4

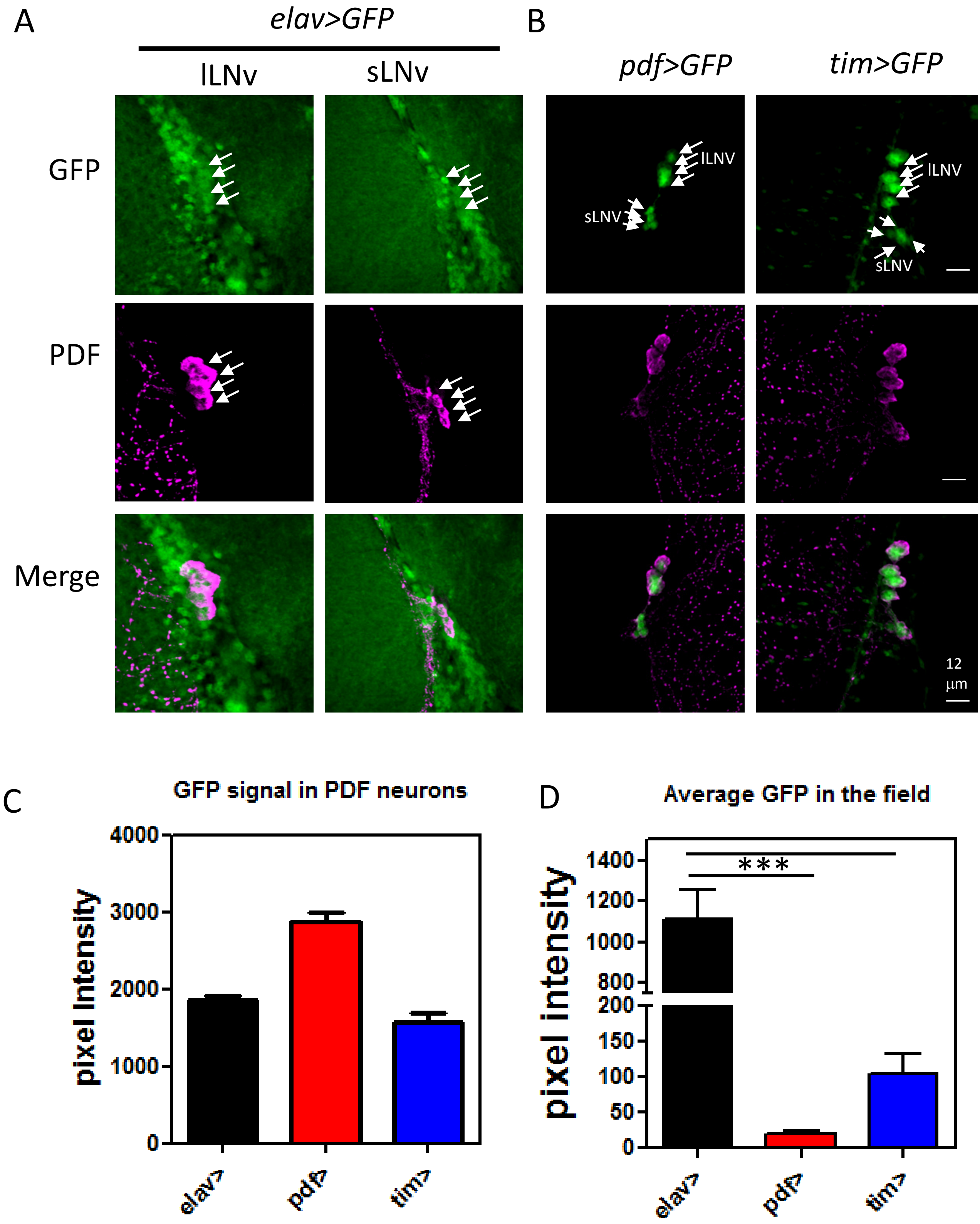
A
elav>GFP
lLNv
sLNv
GFP
PDF
Merge
B
pdf>GFP
tim>GFP
lLNV
sLNV
lLNV
sLNV
12
μm
C
GFP signal in PDF neurons
pixel Intensity
4000
3000
2000
1000
0
elav>
pdf>
tim>
D
Average GFP in the field
pixel intensity
1400
1200
1000
800
200
150
100
50
0
***
elav>
pdf>
tim>

Figure S5

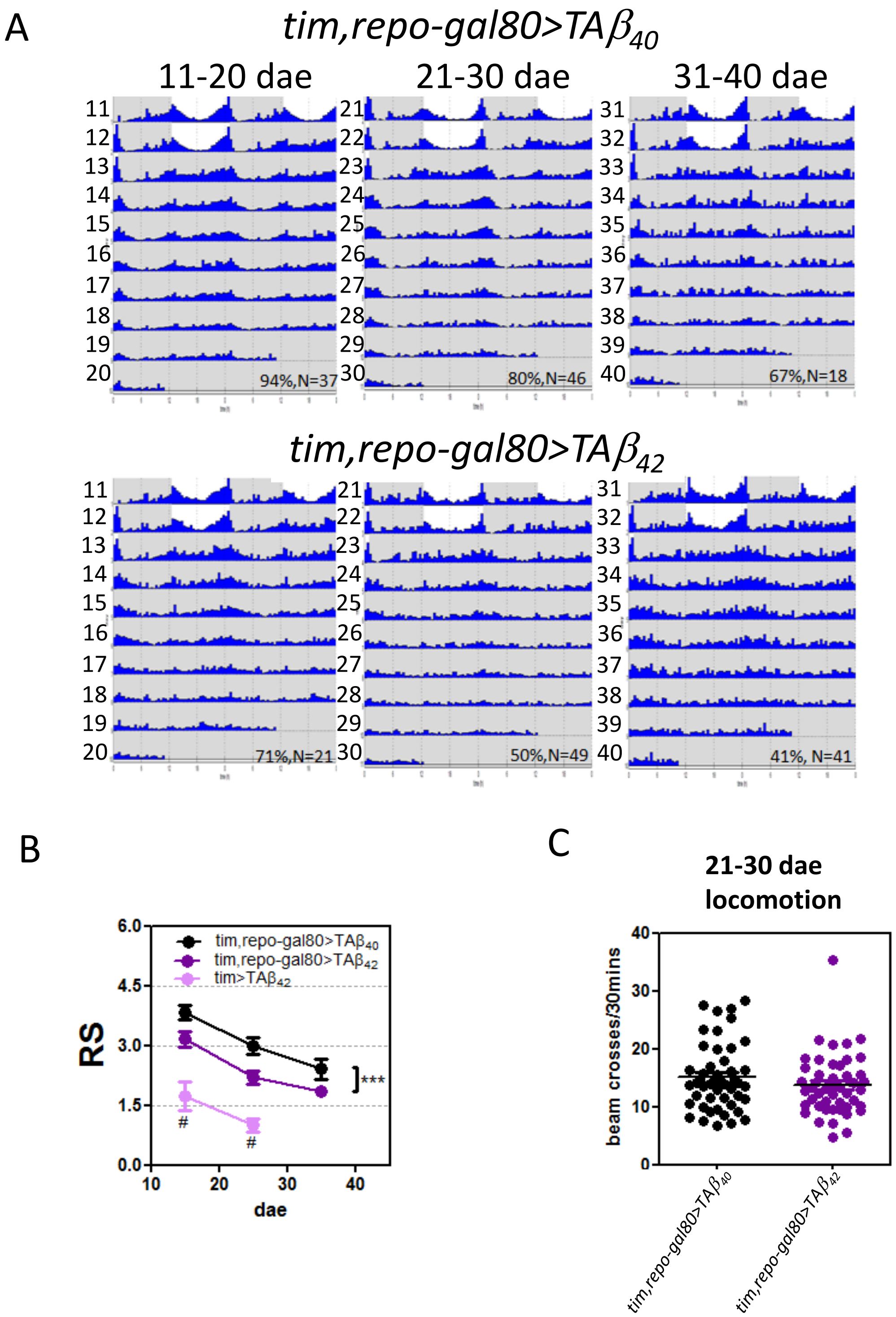

A
tim,repo-gal80>TAβ40
11-20 dae
21-30 dae
31-40 dae
94%,N=37
80%,N=46
67%,N=18
tim,repo-gal80>TAβ42
71%,N=21
50%,N=49
41%, N=41
B
RS
tim,repo-gal80>TAβ40
tim,repo-gal80>TAβ42
tim>TAβ42
***
#
dae
C
21-30 dae
locomotion
beam crosses/30mins
tim,repo-gal80>TAβ40
tim,repo-gal80>TAβ42

Figure S6

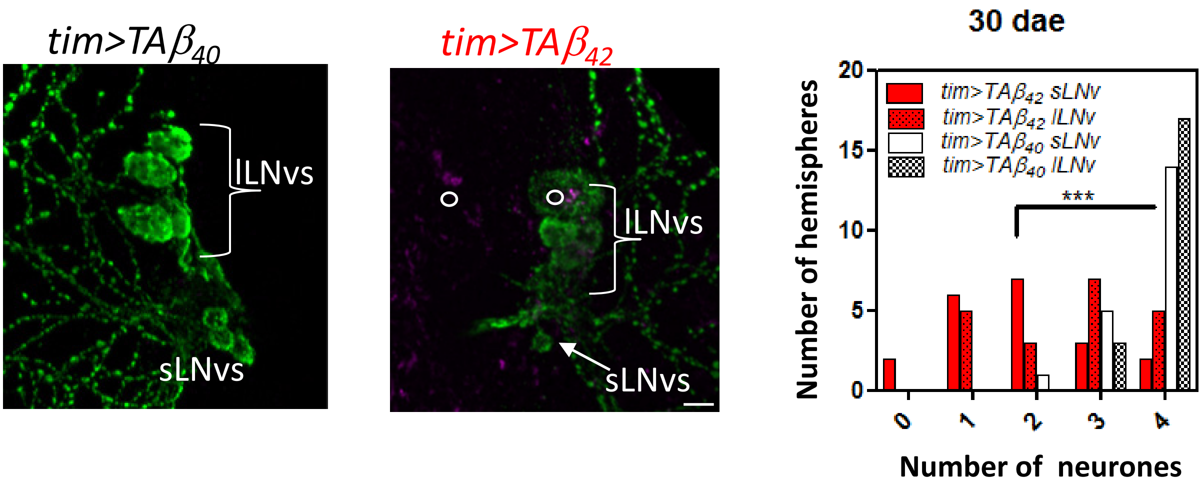
tim>TAβ40
lLNvs
sLNvs
tim>TAβ42
lLNvs
sLNvs
30 dae
tim>TAβ42 sLNv
tim>TAβ42 lLNv
tim>TAβ40 sLNv
tim>TAβ40 lLNv
***
Number of hemispheres
20
15
10
5
0
0
1
2
3
4
Number of neurones

Figure S1. Aβ plaque density in the fly brains does not correlate with the number of clock neurones or with rhythmicity. Representative images of *elav>51D* control (A) and *elav>Aβ42arc* brains (B) show areas that were immunoreactive for Period (green, arrows, DN1s shown only) and Aβ (magenta). A threshold signal intensity in the magenta channel was chosen so that the Aβ positive signal (C, *elav>51D* & D, *elav>Aβ42arc*) faithfully represents the appearance of the raw image. The area above threshold (μm^2) was divided by the total area in the field to calculate “fraction of Aβ positive area”. A significantly higher fraction of the area is Aβ-positive in $A\beta_{42}$arc-expressing fly brains as compared to controls (E, mean±SEM, $p<0.001$ *Student* t-test, the same data set from Figure 3C and D). After five days of circadian behaviour testing, rhythmically robust ($A\beta_{42}$r, RS>1.5, n=3) and arrhythmic ($A\beta_{42}$ar, RS≤1.5, n=6) 37 dae old *elav>*$A\beta_{42}$ flies were re-grouped in separate food vials. Following a further 3 days in LD the brains were dissected at ZT3 (F). Areas of positive Aβ signal (mean±SEM) were quantified and no difference was found between the two groups (*Student t-test*). Furthermore, no correlation (p=0.37) exists between the number of Period positive clock neurones and the density of Aβ plaques (area of Aβ positive) for *elav>*$A\beta_{42}$*arc* (G, 30 dae, ZT22, the same dataset as in Figure 3). Scale bars: 10μm. ZT denotes zeitgeber time with ZT0 indicating dawn and ZT12 dusk during LD cycles.

Figure S2. Robust Period oscillation in clock neurones in behaviourally arrhythmic $A\beta_{42}$arc-expressing fly brains during constant darkness. Period (green ovals) and Aβ (magenta) staining was performed for 28 dae old controls (*elav>51D*) and *elav>*$A\beta_{42}$*arc* flies that have been reared in constant darkness. The fly brains were sampled at the four indicated time points in the second day of constant darkness (CT02, CT08, CT14 and CT20); CT denotes constant time, with CT00 indicating subjective dawn and CT12 subjective dusk. (A) Period and Aβ signals in the indicated clock neurones for *elav>*$A\beta_{42}$*arc* flies and controls at the four time points. The average Period signal in each image was quantified (e.g. panel i, i’ and i”) by measuring the mean pixel intensity (Mi’) in the area within clock neurones in the green channel (i’). Similarly the background pixel intensities (Mi”) in the area adjacent to clock neurones (i”) were also quantified. **Relative Period level** was then calculated by subtracting and normalising the background (Mi’-Mi”/Mi”) and multiplying with the area containing clock neurones (Area.i’) to account for the signal variation in the background and the number of detectable clock neurones at each images (modified from Chen et al., 2011). For time points in which Period staining cannot be identified (e.g. ii), the relative Period level was assigned as zero. (B) Both control (white bars)

and Aβ_{42}arc expressing flies (red bars) showed clear oscillation of Period levels (mean±SEM) across the four time points (non-parametric one-way ANOVA, p<0.001). The numbers of brain hemispheres observed at each time point and genotypes are indicated by the respective bar. Scale bars: 10μm.

Figure S3. Aβ plaque density does not show circadian oscillation. (A) Aβ staining (magenta) was quantified for the Aβ_{42}arc-expressing brain hemispheres shown in Figure S2. No differences in the fraction of Aβ positive area (mean±SEM) were observed at the four indicated time points during constant darkness. (B) There was no difference in the fraction of Aβ positive area (magenta, mean±SEM) between ZT3 and ZT20 during LD cycles in the image stacks of *tim,repo-gal80>TAβ_{42}* fly brains (from Figure 6C). Green: PDF staining. Scale bars: 50μm

Figure S4. Comparing GFP expression in PDF neurones using elav-, pdf- and tim-gal4 driver constructs. Representative images show that UAS-driven GFP intensity (green) was similar for each of the three gal4 drivers used in this study. Quantification was performed by measuring GFP signal within cells expressing endogenous PDF peptide (magenta, arrows, ILNvs and sLNvs) in *elav>GFP* (n=125 neurones, A) and *pdf>GFP* (n=56, B) and *tim>GFP* (n=44, B) male fly brains. Confocal image stacks along frontal-posterior axis were taken to include all PDF neurones. The GFP signal within the cellular outline of individual PDF neurons was measured by ImageJ system (pixel intensity in greyscale, maximum=4096, C). The average GFP signal in a fixed field of 318 μm^2 was calculated for all the stacked images for each genotype (D). The number of fields used to determine the average background GFP staining was 5 for *elav>GFP*, 4 for *pdf>GFP* and 6 for *tim>GFP*. Asterisks mark significant difference as determined by non-parametric one-way ANOVA (***=p<0.001).

Figure S5. Restricting TAβ_{42} expression to clock neurones resulted in intermediate circadian arrhythmicity. (A) Representative actograms are shown for *tim,repo-gal80>TAβ_{42}* (reduced rhythmicity) and control *tim,repo-gal80>TAβ_{40}* at the indicated ages. Total number of flies tested (n) and percentage of rhythmic flies in each genotype (%) are indicated. (B) RS values for *tim,repo-gal80>TAβ_{42}* and *tim,repo-gal80>TAβ_{40}* flies at all tested age groups are plotted. The significance of overall differences in the RS values between the two genotypes was determined by two-way ANOVA (***: p<0.001). Significant differences in the RS values was identified by one-way ANOVA (#: p<0.05) among the four groups of flies *tim,repo-gal80>TAβ_{42}* (11-20 dae and 21-30 dae) and *tim>TAβ_{42}* flies (n=8, 11-20 dae and n=9, 21-30 dae).

The tim(67) gal4 driver line was used in this experiment. (C) No difference in average locomotor activity (i.e., beam crosses) were found between *tim,repo-gal80>TA*β_{42} and *tim,repo-gal80>TA*β_{40} flies aged 21-30 dae (re-analysis from A).

Figure S6. TAβ_{42} expression in clock cells resulted in loss of PDF neurones. Drastic loss of PDF neurons (green) coincided with Aβ positive staining (magenta, circles, both inside and outside of PDF neurones) was found in *tim>TA*β_{42} fly brain (n=20, in brain hemisphere) as compared to *tim>TA*β_{40} flies (n=20). The number of PDF neurones was counted for both sLNvs and lLNvs in the two genotypes. Significant difference determined by χ_2-test was found between the two genotypes for both sLNvs and lLNvs (***: $p<0.001$, see Materials and Methods).

Table S1. Summary of rhythmic luciferase signal from *8.0-luc* flies

age	genotype	R	Sum	R%	Amp
13-17 dae	*elav>51D*	4	12	33%	3.0±0.5
13-17 dae	*elav>* $A\beta_{42}$*arc*	11	12	92%	4.5±0.2
16-20 dae	*elav>51D*	4	12	33%	3.2±0.5
16-20 dae	*elav>* $A\beta_{42}$*arc*	3	12	25%	3.2±0.2
19-23 dae	*elav>51D*	3	12	25%	2.1±0.2
19-23 dae	*elav>* $A\beta_{42}$*arc*	5	12	42%	3.8±0.3
20-24 dae	*elav>51D*	4	12	33%	3.0±0.4
20-24 dae	*elav>* $A\beta_{42}$*arc*	4	12	33%	2.6±0.3
23-27 dae	*elav>51D*	3	12	25%	3.5±0.7
23-27 dae	*elav>* $A\beta_{42}$*arc*	5	12	42%	3.1±0.3
25-29 dae	*elav>51D*	3	12	25%	2.4±0.3
25-29 dae	*elav>* $A\beta_{42}$*arc*	6	12	50%	2.8±0.5
19-23 dae	*tim>T*$A\beta_{40}$	23	28	82%	3.8±0.3
19-23 dae	*tim>T*$A\beta_{42}$	22	26	85%	*2.7±0.2
25-29 dae	*tim>T*$A\beta_{40}$	15	19	79%	2.9±0.1
25-29 dae	*tim>T*$A\beta_{42}$	12	16	75%	2.6±0.7
27-31 dae	*tim>T*$A\beta_{40}$	10	16	63%	3.1±0.5
27-31 dae	*tim>T*$A\beta_{42}$	6	16	38%	3.7±0.4

R: number of flies containing rhythmic luciferase signal (rel-amp error <0.7), Sum: number of fly tested. Amp (mean±SEM): relative amplitude for rhythmic luciferase. Kruskal-Wallis ANOVA statistics with Dunn's Multiple Comparison test are used to determine significant difference in amplitude as compared to age matched controls (*:p<0.01). No difference are found in all pair-wise comparison between *elav>51D* and *elav>Aβ42arc*. A minor difference in amplitude was detected for 19-23 dae old *tim>T*$A\beta_{42}$*/8.0-luc*. This difference is unlikely to cause circadian arrhythmicity because the amplitude is similar to the behaviourally rhythmic control at older ages (c.f. *tim>T*$A\beta_{40}$ and *tim>T*$A\beta_{40}$*/8.0-luc*, Table 1 and *tim>T*$A\beta_{40}$ flies, 25-29 dae, Table S1).
